# Preclinical antivenom-efficacy testing reveals potentially disturbing deficiencies of snakebite treatment capability in East Africa

**DOI:** 10.1371/journal.pntd.0005969

**Published:** 2017-10-18

**Authors:** Robert A. Harrison, George O. Oluoch, Stuart Ainsworth, Jaffer Alsolaiss, Fiona Bolton, Ana-Silvia Arias, José-María Gutiérrez, Paul Rowley, Stephen Kalya, Hastings Ozwara, Nicholas R. Casewell

**Affiliations:** 1 The Alistair Reid Venom Research Unit, Parasitology Department, Liverpool School of Tropical Medicine, Liverpool, Merseyside, United Kingdom; 2 The Institute of Primate Research, National Museums of Kenya, Karen, Nairobi, Kenya; 3 Instituto Clodomiro Picado, Facultad de Microbiología, Universidad de Costa Rica, San José, Costa Rica; 4 County Health Services, County Government of Baringo, Kabarnet, Baringo, Kenya; Institut de Recherche pour le Développement, BENIN

## Abstract

**Background:**

Antivenom is the treatment of choice for snakebite, which annually kills an estimated 32,000 people in sub-Saharan Africa and leaves approximately 100,000 survivors with permanent physical disabilities that exert a considerable socioeconomic burden. Over the past two decades, the high costs of the most polyspecifically-effective antivenoms have sequentially reduced demand, commercial manufacturing incentives and production volumes that have combined to create a continent-wide vacuum of effective snakebite therapy. This was quickly filled with new, less expensive antivenoms, many of which are of untested efficacy. Some of these successfully marketed antivenoms for Africa are inappropriately manufactured with venoms from non-African snakes and are dangerously ineffective. The uncertain efficacy of available antivenoms exacerbates the complexity of designing intervention measures to reduce the burden of snakebite in sub-Saharan Africa. The objective of this study was to preclinically determine the ability of antivenoms available in Kenya to neutralise the lethal effects of venoms from the most medically important snakes in East Africa.

**Methods:**

We collected venom samples from the most medically important snakes in East Africa and determined their toxicity in a mouse model. Using a ‘gold standard’ comparison protocol, we preclinically tested the comparative venom-neutralising efficacy of four antivenoms available in Kenya with two antivenoms of clinically-proven efficacy. To explain the variant efficacies of these antivenoms we tested the IgG-venom binding characteristics of each antivenom using *in vitro* IgG titre, avidity and venom-protein specificity assays. We also measured the IgG concentration of each antivenom.

**Findings:**

None of the six antivenoms are preclinically effective, at the doses tested, against all of the most medically important snakes of the region. The very limited snake polyspecific efficacy of two locally available antivenoms is of concern. *In vitro* assays of the abilities of ‘test’ antivenom IgGs to bind venom proteins were not substantially different from that of the ‘gold standard’ antivenoms. The least effective antivenoms had the lowest IgG content/vial.

**Conclusions:**

Manufacture-stated preclinical efficacy statements guide decision making by physicians and antivenom purchasers in sub-Saharan Africa. This is because of the lack of both clinical data on the efficacy of most of the many antivenoms used to treat patients and independent preclinical assessment. Our preclinical efficacy assessment of antivenoms available in Kenya identifies important limitations for two of the most commonly-used antivenoms, and that no antivenom is preclinically effective against all the regionally important snakes. The potential implication to snakebite treatment is of serious concern in Kenya and elsewhere in sub-Saharan Africa, and underscores the dilemma physicians face, the need for clinical data on antivenom efficacy and the medical and societal value of establishing independent preclinical antivenom-efficacy testing facilities throughout the continent.

## Author summary

Snakebite is one of the most under-researched, under-resourced high morbidty/high mortality NTDs, as reflected by the fact that many of the antivenoms used to treat snakebite victims in sub-Saharan Africa are of uncertain and untested efficacy. This Kenya case study is the first examination of the preclinical efficacy of all available antivenoms to neutralize the venom toxic effects of the most medically important snakes in any region of sub-Saharan Africa. Our results identify serious preclinical efficacy limitations in two of the most commonly used antivenoms, that no single antivenom is effective against all regionally important snakes and that the least effective antivenoms had the lowest IgG concentrations.

It is our aim that Ministry of Health medicine-supply regulators can use this data as evidence to demand more detailed efficacy evidence from manufacturers, and to justify the establishment of national/regional preclinical testing facilities. We hope this publication will also alert physicians treating African snakebite victims to check the efficacy of antivenom in their pharmacies. We have carefully qualified the extent and limitation of the results and of our interpretation of the clinical implications thereof.

## Introduction

Snakebite annually kills over 95,000 people [1] residing in some of the most disadvantaged rural communities [2], and leaves about 300,000 surviving victims with permanent physical disabilities and stigmatising disfigurements. Since it is the most economically-productive and educationally-vulnerable 10–30 year olds that suffer most, snakebite also poses a significant additional socioeconomic burden on these remote, already impoverished communities. Available mortality data clearly indicate that snakebite deaths are greatest in Asia, and particularly in India [1, 3] followed by sub-Saharan Africa (Table 1). The increasing concern over the plight of sub-Saharan African snakebite victims [4, 5, 6] focuses upon the higher case fatality in sub-Saharan Africa than elsewhere (Table 1) and upon the declining availability of effective antivenom to treat snakebite victims.

**Table 1:** Global snakebite incidence and fatality statistics.

Region	Bite Incidences	Deaths	% Fatality
**Sub-Saharan Africa**	420,000	32,000	7.6%
**South Asia**	1,100,000	58,000	4.8%
**Latin America**	129,000	2,300	1.7%
Adapted from ‘high estimate’ data from Kasturiratne et al. [1]			

The crisis in supply of effective and affordable antivenom to treat snakebite victims in sub-Saharan Africa was first reported in 2000 [7], and has since deteriorated. Akin to the late 1990s market failure of the Behringwerke-manufactured antivenom, Sanofi Pasteur had also supplied Africa with one of the most polyspecifically-effective and widely-used antivenoms, FavAfrique, but ceased its manufacture in early 2016 after a more than a decade of commercial disincentives. This latest market failure of effective antivenom particularly affected snakebite-treatment capability in those state, private (mostly city-based) and charity hospitals that could afford this relatively expensive antivenom ($140/vial; [8]). The SAIMR polyvalent antivenom, manufactured by the South African Vaccine Producers Pty (SAVP), was also widely used and recognised to be highly effective–but outside of the Southern Africa Economic Community it has become more expensive ($315/vial, SAVP, personal communication) than FavAfrique was and, also because of low production volumes, become increasingly difficult to source.

**Fig 1:**
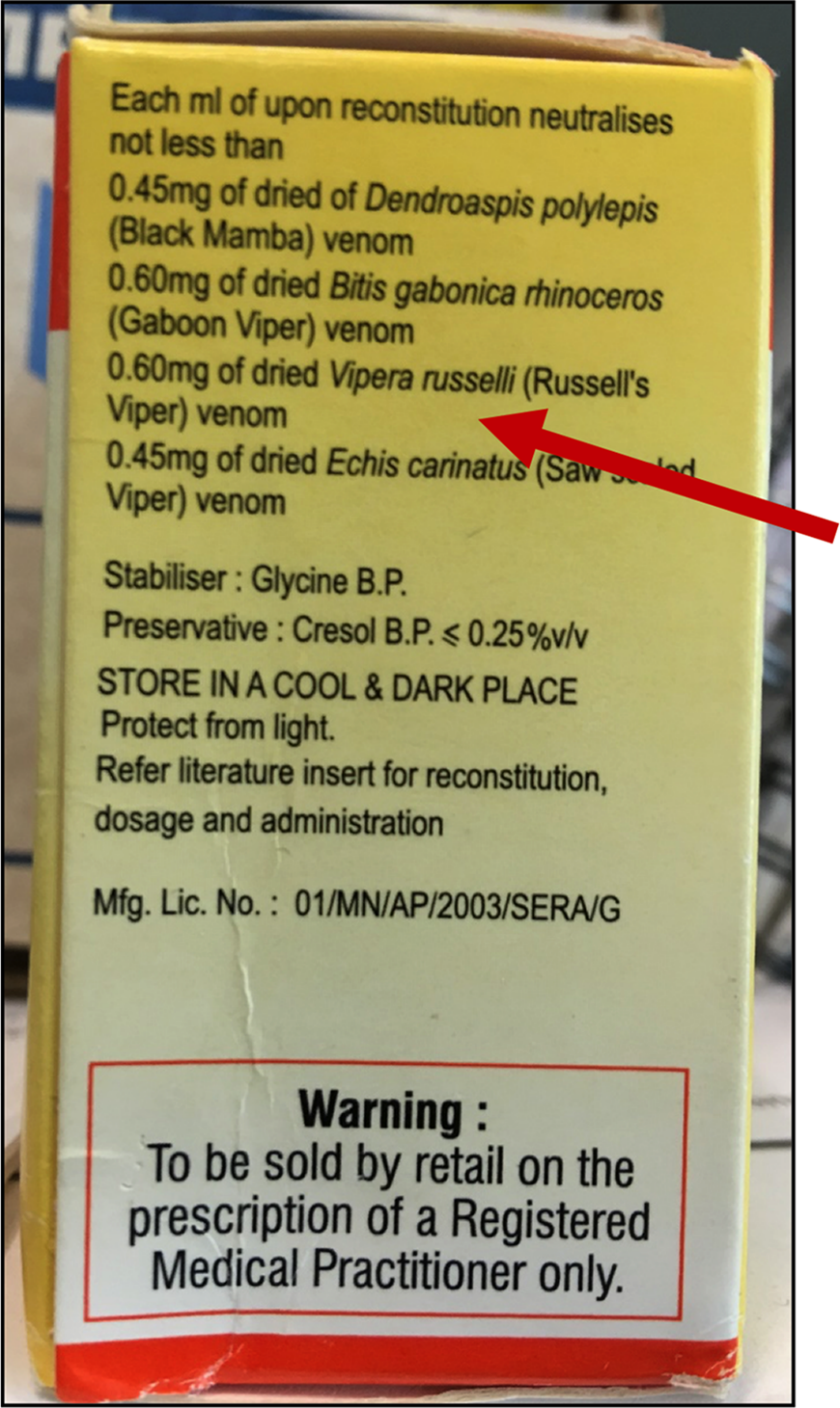
Concerns over an antivenom widely marketed in sub-Saharan Africa. Why are venoms from the Asian Russell’s viper (*Daboia russelii*—incorrectly labelled here as *Vipera russelli*) and saw-scaled viper (*Echis carinatus*) included in its efficacy statement?.

Cognisant presumably of potential commercial opportunities and the public health needs engendered by the snakebite-therapy vacuum in sub-Saharan Africa, several non-Africa based antivenom manufacturers have in the past two decades produced polyspecific antivenoms marketed at costs considerably lower ($18–75) than the FavAfrique or SAIMR antivenoms, and supplied in vastly greater quantities [8]. Superficially, this influx of new, affordable antivenoms into sub-Saharan Africa would seem highly desirable. However, in too many cases and African countries, this has not been the case—because some of these antivenoms have proved dangerously ineffective. Thus, reports from Ghana, Chad and the Central African Republic [9, 10, 11] document an increased case fatality rate (from under 2% to over 12%) following discontinuation of effective antivenoms and introduction of replacement products. In at least one case this was because the antivenom had been manufactured from IgG purified from horses immunised with venoms from Indian snakes–instead of venoms from African snakes [12]. Antivenom efficacy is predominantly restricted to snakes whose venoms were used in manufacture [13]–because the highly snake species-specific protein composition of venom dictates an equally specific IgG response in the immunised horses/sheep. Thus, the greater the biogeographic difference between the venom/s used in antivenom manufacture and the venom injected into the snakebite patient, the weaker the efficacy of the antivenom. For this reason, antivenom manufacturers are required to preclinically test and state the snake species for which their product is effective. Fig 1 evidences another antivenom marketed specifically for Central Africa but clearly inappropriately manufactured with venoms from Asian vipers.

There are very few published reports on the clinical effectiveness of the several antivenoms in current use in sub-Saharan Africa. Preclinical efficacy data is therefore the only information available to physicians and government purchasers to decide which antivenom to use/purchase. However, many, perhaps the majority, of sub-Saharan African countries do not apparently subject newly-imported antivenoms to independent preclinical efficacy and safety testing, and clinicians and purchasers *per-force* base clinical use/purchase decision making upon manufacture-stated efficacy statements. The above reports of antivenom ineffectiveness and rising case fatalities, seemingly throughout much of sub-Saharan Africa, demonstrate this trust can be misplaced. There is therefore an urgent need to establish independent preclinical antivenom-efficacy testing facilities and expertise in sites throughout sub-Saharan Africa.

With substantive new funding, the Liverpool School of Tropical Medicine has partnered with colleagues in Kenya, Nigeria and Cameroon to form the African Snakebite Research Group and established ‘Snakebite Research and Intervention Centres’ (SRIC) in each of these countries. Our remit includes improving the (i) availability of effective snakebite treatment in rural remote hospitals in greatest need and (ii) access to treatment for snakebite victims. To ensure this new programme is equipped with effective antivenom, and to provide the host government with independent antivenom-efficacy information, we purchased a vial of as many different antivenom brands as available from local pharmacies and preclinically tested their efficacy against venoms of the most medically important snakes in the region.

This first report from the African Snakebite Research Group emanates from Kenya-SRIC activity and demonstrates, for the first time in East Africa, that there is substantial variation in the preclinical efficacy of the available antivenoms against the lethal effects of venoms from black mambas, spitting and non-spitting cobras, puff adders and saw-scaled vipers–and that no one antivenom is preclinically effective at the doses tested, against all these life-threatening snake venoms.

## Materials and methods

### Antivenoms

The antivenoms used in this study are described in detail in Table 2 and were acquired from a commercial pharmacy in Nairobi, except the SAIMR antivenoms that were donated to the first author from expired stocks held by Public Health England and had 2012 expiry dates. We were unsuccessful in purchasing one of the ASNA antivenoms manufactured by Bharat Serums and Vaccines Ltd that we had seen in a rural hospital in Kenya. All the antivenoms are manufactured as F(ab')_2_ fragments of IgG, and for clarity to non-specialist readers we have used the term IgG to describe these antivenoms.

**Table 2:** Information upon each antivenom described by the product insert.

Brand	Manufacturer	Characteristics	Stated Efficacy
**‘Test’ Antivenom** Polyvalent Snake Venom Antiserum—(PAN AFRICA)	Premium Serums and Vaccines, PVT. Ltd, Quthbullapur, India	Expiry Date: 01/2018 Batch #:062003 - Lyophilised - Equine F(ab’)_2_ - Immunised with venoms opposite	Each ml neutralises over 25 venom LD_50_ for: *Bitis arietans*, *B*. *gabonica*, *B*. *g*. *rhinoceros*, *B*. *nasicornis*, *Echis ocellatus*, *E*. *leucogaster*, *E*. carinatus*, *Naje haje*, *N*. *melanoleuca*, *N*, *nigricollis*, *N*. *mossambica*, *Dendroaspis polylepis*, *D*. *viridis*, *D*. *jamesoni*, *D*. *angusticeps*.
**‘Test’ Antivenom** Snake Venom Antiserum (African)	VINS Bioproducts Ltd, Telangana, India	Expiry Date: 04/2020 Batch #:07A515003 - Lyophilised - Equine F(ab’)_2_ - Immunising venoms not stated	Each ml neutralises over 20 venom LD_50_ for: *B*. *arietans*, *B*. *gabonica*, *E*. *ocellatus*, *E*. *leucogaster*, *N*. *haje*, *N*. *melanoleuca*, *N*, *nigricollis*, *D*. *polylepis*, *D*. *viridis*, *D*. *jamesoni*.
**‘Test’ Antivenom** Inoserp PANAFRICAIN	INOSAN Biopharma, SA Madrid, Spain	Expiry Date: 11/2018 Lot #: 51T11003 - Lyophilised - Equine F(ab’)_2_ - Immunising venoms not stated	Each vial contains no less than 500 units of neutralisation (LD50) for venoms of *E*. *ocellatus*, *B*. *arietans*, *N*, *nigricollis* and *D*. *polylepis* and recommended for treating victims of *E*. *leucogaster*, *E*. *pyramidum*, *B*. *gabonica*, *B*. *g*. *rhinoceros*, *D*. *viridis*, *D*. *angusticeps*, *D*. *jamesoni*, *N*. *haje*, *N*.*pallida*, *N*. *melanoleuca*, *N*. *nivea*, *N*. *katiensis*.
**‘Test’ Antivenom** FavAfrique	Sanofi Pasteur SA, Lyon, France	Expiry Date: 06/2016 Lot #: K8453-1 - Liquid - Equine F(ab’)_2_ - Immunising venoms not stated	Each ml neutralises over 25 venom LD_50_ for: *Bitis arietans*, *B*. *gabonica*, *E*. *ocellatus*, *E*. *leucogaster*, *D*. *polylepis*, *D*. *viridis*, *D*. *jamesoni and* neutralises over 20 venom LD_50_ for *N*. *haje*, *N*. *melanoleuca*, *N*, *nigricollis*,
**‘Gold Standard’ Antivenom** SAIMR Polyvalent Snake Antivenom	South African Vaccine Producers (PTY) Ltd, Gauteng, South Africa	Expiry Date: 08/2012 Lot #: X02646—Liquid - Equine F(ab’)_2_ - Immunised with venoms of *B*. *arietans*, *B*. *gabonica*, *D*. *polylepis*, *D*. *jamesoni*, *D*. *angusticeps; N*. *nivea*, *N*. *melanoleuca*, *N*. *annulifera*, *N*. *mossambica*, *Hemachatus haemachatus*	Effective against venoms of all Rinkhals, mambas, cobras and vipers likely to cause life-threatening envenomation in Southern and Central Africa.
**‘Gold Standard’ Antivenom** SAIMR ECHIS CARINATUS Antivenom	South African Vaccine Producers (PTY) Ltd, Gauteng, South Africa	Expiry Date: 02/2012 Lot #: X00447—Liquid - Equine F(ab’)_2_ - Immunised with venoms from *E*. carinatus*/ocellatus (and perhaps from *E*. *pyramidum* species)	Neutralises the venom of *E*. *ocellatus* and has paralogous potency against venoms of *E*. *coloratus* and two *Cerastes* species.

*E*. carinatus*—this nominal previously covered all *Echis* saw-scaled vipers. The genus has since been split into several species, with *E*. *carinatus* sub-species (*E*. *c*. *carinatus*, *E*. *c*. *sochureki*) only found in the Middle East and the Asian subcontinent The inclusion here of *“ECHIS CARINATUS”* in the brand name does not therefore refer to Asian/Middle East saw-scaled vipers.

The comparative preclinical efficacy of the ‘test’ antivenoms was conducted before their respective expiry dates. We were unable to purchase the SAIMR polyvalent and ECHIS CARINATUS monovalent ‘gold standard’ antivenom in Kenya and therefore used 2012-expired vials donated to us from Public Health England. SDS-PAGE profiling of these antivenoms (Supplementary S3 Fig) reveal that the IgG in these SAIMR antivenoms possess the same structural integrity as IgG from the ‘in date’ test antivenoms. Further validation of using these expired SAIMR antivenoms for this study is provided by the comprehensive binding of venom proteins by IgG in these antivenoms.

### Snake venoms

East Africa is resident to multiple medically important vipers, elapids and colubrids. For this analysis, we selected venoms from the most relevant representative species of each genus. Venom was extracted from over four specimens of wild-caught puff adders (*Bitis arietans*, Kenya); saw-scaled vipers (*Echis pyramidum leakeyi*, Kenya); black mambas (*Dendroaspis polylepis*, Tanzania); Egyptian cobras (*Naja haje*, Uganda); black-necked spitting cobras (*N*. *nigricollis*, Tanzania) and red spitting cobras (*N*. *pallida*, Kenya) maintained in the Liverpool School of Tropical Medicine herpetarium (a UK Home Office accredited and inspected animal research facility). Freshly collected venom was snap frozen, lyophilised and stored as a powder at 4°C prior to reconstitution in phosphate-buffered saline (PBS). The same batches of these venoms were used for each of the analyses below to provide cross-experiment continuity.

### Preclinical venom toxicity and antivenom efficacy assays

#### Venom lethality in mice

As an essential prerequisite to assessing antivenom efficacy, we determined the median murine lethal dose (LD_50_) for each of the snake venoms using WHO-recommended protocols [13]. Briefly, groups of five male CD-1 mice (18-20g) received an intravenous (iv) tail injection of varying doses of venom in 100μl PBS (0.12 M NaCl, 0.04 M phosphate, pH 7.2) and, 24 hours later, the number of surviving mice in each group was recorded. The venom LD_50_ (the amount of venom that kills 50% of the injected mice) and 95% confidence limits of each snake species was calculated by probit analysis [13]. The venom LD_50_ assays were performed at the Instituto Clodomiro Picado (University of Costa Rica) using protocols approved by the Institutional Committee for the Use of Laboratory Animals (CICUA) of the University of Costa Rica (project 82–08).

#### Antivenom neutralisation of venom-induced lethality in mice

We used the median antivenom effective dose (ED_50_) assay [13] to determine the venom-neutralising effectiveness of (i) the SAIMR polyvalent ‘gold standard’ antivenom against venoms from *B*. *arietans*, *D*. *polylepis*, *N*. *haje*, *N*. *nigricollis* and *N*. *pallida* and (ii) the SAIMR ECHIS CARINATUS monovalent ‘gold standard’ antivenom against venom from *E*. *p*. *leakeyi*. Thus, various doses of the antivenom were mixed with 5 venom LD_50_s (2.5 venom LD_50_s for *N*. *nigricollis*; a dose of venom that is lethal to 100% of mice–a requirement of the assay design) and a final volume of 200μl prepared with PBS. The venom/antivenom mixtures were incubated at 37°C for 30 minutes and iv injected into the tail vein of groups of 5 CD-1 mice and, 7 hours later, the number of surviving mice in each group was recorded. The median effective dose (the least amount of antivenom required to prevent death in 50% of mice injected with 2.5 or 5 venom LD_50_s) and 95% confidence limits were calculated using probit analysis [13].

#### Ethics statement

The venom LD_50_ assays were performed at the Instituto Clodomiro Picado (University of Costa Rica) using protocols approved by the Institutional Committee for the Use of Laboratory Animals (CICUA) of the University of Costa Rica (project 82–08). All antivenom efficacy animal experiments were conducted using protocols approved by the (i) Liverpool School of Tropical Medicine, and University of Liverpool Animal Welfare Ethical Review Boards and (ii) the UK Home Office under the Animals (Scientific procedures) Act 1986, that incorporate analgesic, humane end point and dose-staging refinements to reduce the extent and duration of pain, harm and distress of the lowest possible number of experimental mice.

### In vitro immunological assays

We employed routine protocols in our laboratory [14] to measure the IgG titre, avidity, venom protein-specificity and protein (IgG) concentration/ml antivenom to provide a detailed immunological profile of the ‘gold standard’ and ‘test’ antivenoms.

#### Serial dilution ELISA

ELISA wells (96 well plates) were coated with 100ng of venom prepared in carbonate buffer, pH 9.6 and incubated at 4°C overnight. After washing with TBST (0.01 M Tris-HCL pH 8.5; 0.15 M NaCl; 1% Tween 20), the venom-coated plates were incubated at room temperature (RT; typically 22–24°C in our laboratory) for 3 hours with 5% non-fat milk in TBST to ‘block’ non-specific reactivity. Antivenoms were added to the washed plates as 1:5 serial dilutions (in PBS; in triplicate) starting with an initial dilution of 1:100 and incubated overnight at 4°C. The plates were washed with TBST and secondary horseradish peroxidise-conjugated rabbit anti-horse IgG (1:1,000; Sigma, UK) added for 3 hours at RT, when unbound antibody was removed by washing with TBST. The results were determined by addition of substrate (0.2% 2,2/-azino-bis (2-ethylbenzthiazoline-6-sulphonic acid) in 0.05 M citric acid buffer, pH 4.0 containing 0.015% hydrogen peroxide (Sigma, UK) for 15 minutes and optical density (OD) measured at 405 nm.

#### Relative avidity ELISA

This assay was performed as above except that the antivenom IgGs were diluted to a single concentration of 1:10,000, incubated overnight at 4°C, washed with TBST and the chaotrope, ammonium thiocyanate (NH_4_SCN), added to the wells in a range of concentrations (0–8 M) for 15 minutes. Plates were washed, and all subsequent steps were the same as the serial dilution ELISA assay.

#### SDS-PAGE and immunoblotting

The lyophilised snake venoms were reconstituted to 1mg/ml in reduced protein loading buffer, boiled (5 mins) and 10μg added to a 15% SDS-PAGE gel and fractionated under 200 volts and the resultant proteins visualised by staining with Coomassie Blue R-250. For immunoblotting, the 15% gels were electro-blotted onto 0.45 μm nitrocellulose membranes using the manufacturer’s protocols (Trans Blot Turbo (13A, 25V, 7 mins), Bio-Rad, UK). Following confirmation of successful protein transfer by reversible Ponceau S staining, the membranes were incubated overnight in blocking buffer (5% non-fat milk in TBST), followed by six washes of TBST over 90 minutes and incubation overnight with antivenoms diluted 1:5,000 in blocking buffer. Blots were washed as above, then incubated for 2 hours with horseradish peroxidise-conjugated rabbit anti-horse IgG secondary antibody (1:2,000 dilution) before a final wash with TBST and visualisation after the addition of DAB substrate (50 mg 3,3-diaminobenzidine, 100 ml PBS and 0.024% hydrogen peroxide; Sigma, UK).

#### Antivenom protein content

The amount of protein (IgG) in each vial of antivenom has a critical bearing upon efficacy and is very rarely stated in the manufacturers’ product inserts. To test this and avoid potential confounding effects of antivenom manufacturers using different, often unstated, excipients, each antivenom was diluted 1/500 in PBS before protein concentration was measured, in triplicate using a NanoDrop 1000 spectrophotometer (Thermo Scientific). Concentrations were determined using the NanoDrop Protein A280 method using the in-built IgG mass extinction coefficient. Controls of equine IgG (Sigma, UK) at known concentrations (250, 500, 750 and 1,000 μg/ml) were used to ensure accurate equipment functionality.

## Results

### Establishing the toxicity of the venoms of East African snakes

#### The venom lethal dose (LD_50_) of the East African snakes

This analysis determines the amount of venom that kills 50% of iv injected mice (5/dose group) and is described for the East African snake venoms in Table 3. The venoms of the black mamba, Egyptian cobra and red spitting cobra exert a substantially greater lethal toxicity (upon mice) than venoms of the puff adder, saw-scaled viper and black-necked spitting cobra.

**Table 3:** The murine venom lethal dose (LD_50_) of the East African snakes. The venom LD_50_ is provided as μg venom per mouse and as μg venom per gram body weight as both metrics are used in the literature.

Common name	Snake Species (Origin)	Venom lethal dose LD_50_ μg/mouse (95% CI)	Venom lethal dose LD_50_ μg/g body weight (95% CI)
Puff adder	*Bitis arietans* (Kenya)	19.55 (14.76–27.40)	1.03 (0.78–1.44)
Saw-scaled viper	*Echis pyramidum leakeyi* (Kenya)	16.00 (10.22–25.43)	0.84 (0.54–1.33)
Black-necked spitting cobra	*Naja nigricollis* (Tanzania)	24.40 (17.64–30.78)	1.28 (0.93–1.62)
Red spitting cobra	*Naja pallida* (Kenya)	9.29 (3.76–13.23)	0.49 (0.20–0.70)
Egyptian cobra	*Naja haje* (Uganda)	8.15 (6.55–9.89)	0.43 (0.35–0.52)
Black mamba	*Dendroaspis polylepis* (Tanzania)	6.17 (3.09–10.20)	0.32 (0.16–0.54)

### Establishing the venom-neutralising efficacy of the ‘gold standard’ SAIMR antivenoms

We used the WHO-recommended antivenom effective dose (ED_50_) assay, which measures the amount of antivenom required to prevent venom-induced lethality in 50% of mice (5/dose group) injected with venom/antivenom mixtures. To assess the efficacy of the SAIMR ‘gold standard’ polyvalent and monovalent antivenoms, we determined the ED_50_ dose of (i) the SAIMR ECHIS CARINATUS monovalent antivenom against only the saw-scaled viper venom and (ii) the SAIMR polyvalent antivenom against venoms of the black mamba, the Egyptian, red and black-necked spitting cobras and the puff adder (Table 4).

#### The antivenom effective dose (ED_50_) of the SAIMR ECHIS CARINATUS antivenom against the East African saw-scaled viper venom

The ED_50_ dose of this saw-scaled viper-specific (monovalent) antivenom against 5x LD_50_s of the Kenyan *E*. *p*. *leakeyi* venom was determined as 17.56 μl (Table 4). This highly effective venom-neutralising dose is consistent with previous preclinical assays of other *Echis* species-specific antivenoms [14, 15, 16]. We did not test the efficacy of the SAIMR polyvalent antivenom against *E*. *p*. *leakeyi* venom because the latter was not included in its stated efficacy statement (venom from this group of snakes is not used as part of the immunising mixture, Table 2).

#### The antivenom effective dose (ED_50_) of the SAIMR polyvalent antivenom against the other East African snake venoms

This antivenom was tested against 5x venom LD_50_ volumes for all the venoms except for the black spitting cobra, *N*. *nigricollis*, for which we were forced to use 2.5x venom LD_50_ volumes because the 5x LD_50_ volume (120 μl) of the venom restricted the amount of antivenom we could administer (the total iv injection volume is restricted to 200 μl). The ED_50_ results of this analysis are presented in Table 4. To facilitate efficacy comparison to the ‘test’ antivenoms, we have added the volume of these SAIMR antivenoms predicted to protect 100% (2xED_50_ –calculated by doubling the ED_50_ volumes) of the mice from the lethal toxic effects of the East African venoms. We experimentally confirmed that these 2xED_50_ doses protected 100% of mice injected with this venom/antivenom mixture.

The SAIMR polyvalent antivenom neutralised the murine lethal effects of all the venoms, and exhibited a substantially greater neutralising potency against the puff adder and black mamba venoms than against venoms from all the cobra species (Table 4). This is not unexpected because these cobra venoms are not used as immunogens in the manufacture of the SAIMR polyvalent antivenom (venom immunogens are from the following related cobra species: Mozambique spitting cobra (*N*. *mossambica*), the cape cobra (*N*. *nivea*), the forest cobra (*N*. *melanoleuca*) and the banded Egyptian cobra (*N*. *annulifera*). This reinforces that the efficacy of an antivenom is greatest to snakes whose venom was used in manufacture and can be somewhat effective against snakes whose venoms contain cross-reactive venom proteins (typically congeners). Importantly, the clinical implication of this preclinical data is that treatment of human snakebite victims may require snake species-distinct doses of the SAIMR polyvalent antivenom.

**Table 4:** The volume of the ‘gold standard’ SAIMR antivenoms that protect 50% and 100% (the ED_50_ and 2x ED_50_) of the mice from the lethal toxicity of the East African snake venoms.

Snake Common name	Snake Species	Venom LD_50_ dose (μg)	The volumes (μl) of SAIMR antivenoms that protect 50% and 100% (the calculated ED_50_ and 2xED_50_ respectively) of the mice from the lethal toxicity of the venoms		
				SAIMR Polyvalent	SAIMR ECHIS CARINATUS
Puff adder	*B*. *arietans*	5x (97.8)	ED_50_	21.07 (18.8–23.5)	
			2xED_50_	42.14	
Saw-scaled viper	*E*. *p*. *leakeyi*	5x (80)	ED_50_		17.56 (15.1–19.3)
			2xED_50_		35.12
Black-necked spitting cobra	*N*. *nigricollis*	2.5x (61)	ED_50_	69.17 (61.7–73.4)	
			2xED_50_	138.34	
Red spitting cobra	*N*. *pallida*	5x (46.5)	ED_50_	73.52 (51.8–84.6)	
			2xED_50_	147.04	
Egyptian cobra	*N*. *haje*	5x (40.8)	ED_50_	71.00 (69.2–73.3)	
			2xED_50_	142.0	
Black mamba	*D*. *polylepis*	5x (30.85)	ED_50_	13.82 (11.8–15.5)	
			2xED_50_	27.64	

### Determining the venom-neutralising efficacy of the ‘test’ antivenoms against the East African snake venoms

We assigned the SAIMR antivenoms as the ‘gold standard’ comparators because of their sub-Saharan African clinical effectiveness [17] and because, unlike FavAfrique, these antivenoms are likely to be available for the foreseeable future.

We elected a ‘gold standard comparison’ experimental design to achieve our objective instead of 24 conventional ED_50_ assays (4 ‘test’ antivenoms tested against 6 venoms), which would have required a minimum of 600 mice (25 mice/experiment). Instead, we tested the extent to which the ‘test’ antivenoms prevented the death of mice (5/dose group) injected with the venom/antivenom mixtures at volumes equivalent to half (0.5x), equal (1x) or, where appropriate, 2.5-fold more (2.5x) the volume of the SAIMR antivenoms that impart 100% survival of the mice (calculated by doubling the ED_50_ dose volume). Thus, 100% survival of mice injected with 0.5x volume of a ‘test’ antivenom indicates a higher venom-neutralising efficacy than the SAIMR ‘gold standard’ antivenoms, and a test antivenom providing less than 100% efficacy at 1x volumes would be deemed less dose-effective than the ‘gold standard’. Failure of an antivenom to neutralise the lethal effects of a venom at 2.5x volumes in 100% of mice would raise serious concerns as to the potential clinical efficacy of that product.

**Fig 2:**
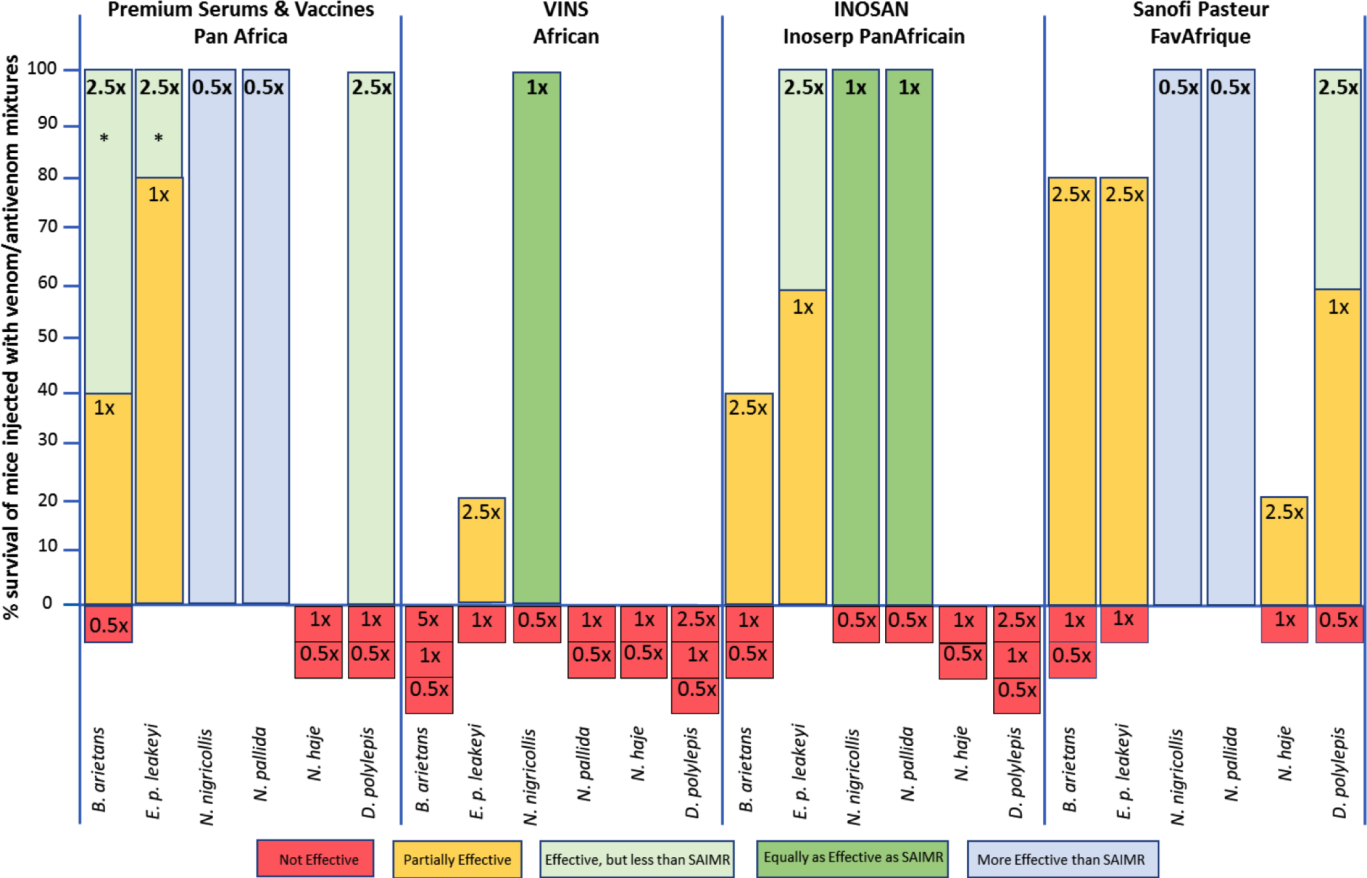
Variant efficacy of the ‘test’ antivenoms against the lethal effects of the six East African venoms in a mouse model. The percent survival of mice injected with lethal doses of venom mixed with the ‘test’ antivenoms in volumes equivalent to half (0.5), the same (1x) or 2.5-fold more (2.5x) of the 100% effective volume of the SAIMR ‘gold standard’ antivenoms was used to identify whether the ‘test’ Premium Serums & Vaccines, VINS, INOSAN and Sanofi Pasteur antivenom were ineffective (red bar), partially effective (yellow bar), effective but requiring a higher dose of antivenom than the SAIMR products (light green bar), an equivalent dose efficacy as the SAIMR products (dark green bar) or a superior dose efficacy than the SAIMR antivenoms (blue bar). The test venoms were East African puff adders (*B*. *arietans*; 97.8 μg*)*, saw-scaled vipers (*E*. *p*. *leakeyi*; 80.5 μg), black necked spitting cobras (*N*. *nigricollis*; 61.0 μg), red spitting cobras (*N*. *pallida*; 46.5 μg), Egyptian cobras (*N*. *haje*; 40.8 μg) and black mambas (*D*. *polylepis*; 30.8 μg). Please refer to Supplementary S1 Table for details on antivenom volumes, amounts of antivenom in mg, snake geographical origin and venom LD_50_ dose used for each experimental group. * Mice died from the high density of antivenom/venom complexes that precipitate out of solution, not from venom-induced effects. With experience, the symptoms of this cause of death can be readily distinguished from that caused by venom. This occurs occasionally in murine preclinical testing as a consequence of the 30 minute, 37°C incubation of the venom/antivenom mixture prior to injection. It likely has no clinical relevance, but can obfuscate preclinical results.

This protocol enabled us to comprehensively analyse the efficacy, compared to a gold standard, of several antivenoms against several venoms using only 40% of the number of mice had we used conventional ED_50_ testing (Fig 2). It is important to note that, for the sake of clarity for non-specialist readers, we have described the efficacy of the ‘test’ antivenoms relative to 0.5x, 1x or 2.5x volumes of the SAIMR antivenom that protect 100% of mice. This differs from the more conventional description as ED_50_ volumes (the volume of antivenom that protects 50% of the venom/antivenom injected mice), and antivenom preclinical testing practitioners are referred to Supplementary S1 Table that depicts the same data in Fig 2 in the context of antivenom volumes (μL) and amounts (mg).

#### Efficacy comparison of the ‘test’ antivenoms with the SAIMR ECHIS CARINATUS monovalent gold standard antivenom against the East African saw-scaled viper (*E*. *p*. *leakeyi*) venom

Since it was highly unlikely that any of the polyvalent ‘test’ antivenoms would possess an equal *E*. *p*. *leakeyi* venom-neutralising efficacy as the monovalent SAIMR ECHIS CARINATUS antivenom, we tested these antivenoms at 1x and 2.5x volumes of the ‘gold standard’ (Fig 2).

The Premium Serums & Vaccines and INOSAN antivenoms were the most effective ‘test’ antivenoms at neutralising the lethal effects of *E*. *p*. *leakeyi* venom. However, they both required more the twice the amount of IgG (88 μl) than the SAIMR ECHIS CARINATUS monovalent antivenom (35 μl) to provide 100% protection. This is not unexpected because monovalent antivenoms are typically more dose-effective than polyvalent antivenoms [15]. The 80% survival of mice given 35 μl of the Premium Serums & Vaccines antivenom suggests that, in the absence of the SAIMR ECHIS CARINATUS antivenom, this product (followed by INOSAN) would be most effective at treating *E*. *p*. *leakeyi* envenoming. The Sanofi Pasteur, and particularly the VINS, antivenoms exhibited the weakest efficacy against *E*. *p*. *leakeyi* venom.

#### Efficacy comparison of the ‘test’ antivenoms with the SAIMR polyvalent gold standard antivenom against venoms of the puff adder (*B*. *arietans*), the spitting cobras (*N*. *nigricollis* and *N*. *pallida*) and the neurotoxic Egyptian cobra (*N*. *haje*) and black mamba (*D*. *polylepis*)

None of the ‘test’ antivenoms were as dose-effective as the SAIMR polyvalent at neutralising the lethal effects of the *B*. *arietans*, *N*. *haje* and *D*. *polylepis* venoms (Fig 2 and Supplementary S1 Table). This was a serious concern because of the frequency and severity of envenoming by these snakes [17]. We therefore repeated the experiment with 2.5x volumes of the SAIMR polyvalent antivenom (we were however unable to test *N*. *haje* venom/antivenom combinations at this antivenom dose because it would have required 355 μl of antivenom and 40 μl of venom and our iv injection volume is restricted to 200 μl). The Premium Serums & Vaccines and Sanofi Pasteur antivenoms were completely effective against the *D*. *polylepis* venom at volumes 2.5x higher (70 μl) than the SAIMR polyvalent antivenom. The 2.5x dose of the Premium Serums & Vaccines antivenom provided 100% protection to the *B*. *arietans* venom, and improved the efficacy of the Sanofi Pasteur antivenom to 80%. This tactic failed to impart any degree of anti-*D*. *polylepis* venom efficacy for the VINS and INOSAN antivenoms, and only resulted in partial neutralisation of *B*. *arietans* venom by the INOSAN product.

The Premium Serums & Vaccines and Sanofi Pasteur antivenoms were more effective, and the INOSAN product as effective as the SAIMR polyvalent in neutralising the lethal effects of the *N*. *nigricollis* and *N*. *pallida* spitting cobra venoms. This is not unexpected because these venoms were not used in the manufacture of the SAIMR polyvalent antivenom (which used *N*. *mossambica* and *H*. *haemachatus* venoms; southern African species that spit venom) but serves to emphasise that the snake species-efficacy and dose-efficacy of an antivenom is critically dependent upon the immunising venoms.

In summary, comparing the various ‘test’ antivenoms to the ‘gold standards’ and to one another demonstrated that the Premium Serums & Vaccines antivenom was more effective against the viper venoms than the Sanofi Pasteur antivenom, but the reverse was true for the neurotoxic *N*. *haje* and *D*. *polylepis* venoms. The INOSAN antivenom achieved parity with the efficacy of the ‘gold standard’ only against venoms of the spitting cobras (but see also results section F). The VINS antivenom exhibited the weakest polyspecific neutralisation of venom-induced lethality. This data clearly demonstrates that there exists substantial variation in the dose-efficacy of each antivenom to the different snake venoms (Fig 2 and Supplementary S1 Table).

The fact that none of the six antivenoms is preclinically effective against all the East African snake venoms was of greatest concern. As stated earlier, the polyspecific venom-neutralising efficacy of a polyvalent antivenom is fundamentally dependent upon the venoms used to immunise the horses, and the proportional representation of the venom from each snake species/genus in the immunizing mixture. Other factors include the quality of the venom, the adjuvant used to promote seroconversion, the venom-dose and frequency of immunisation and the health of the animals [18].

Only three antivenom manufacturers stated the snake species whose venom was used for immunisation and none described the geographic origin of the snakes (Table 2). It was therefore possible that the distinct East African venom-neutralising efficacies of the six antivenoms was a consequence of immunisation with venoms from snakes of significantly different genetic/geographic origin. To investigate this, we conducted a series of *in vitro* immunological analyses to determine the venom species-specific IgG titre, avidity and specificity of the ‘test’ and ‘gold standard’ antivenoms.

### Comparison of antivenom F(ab')_2_ IgG titre to the East African snake venoms by an IgG titration ELISA assay

**Fig 3:**
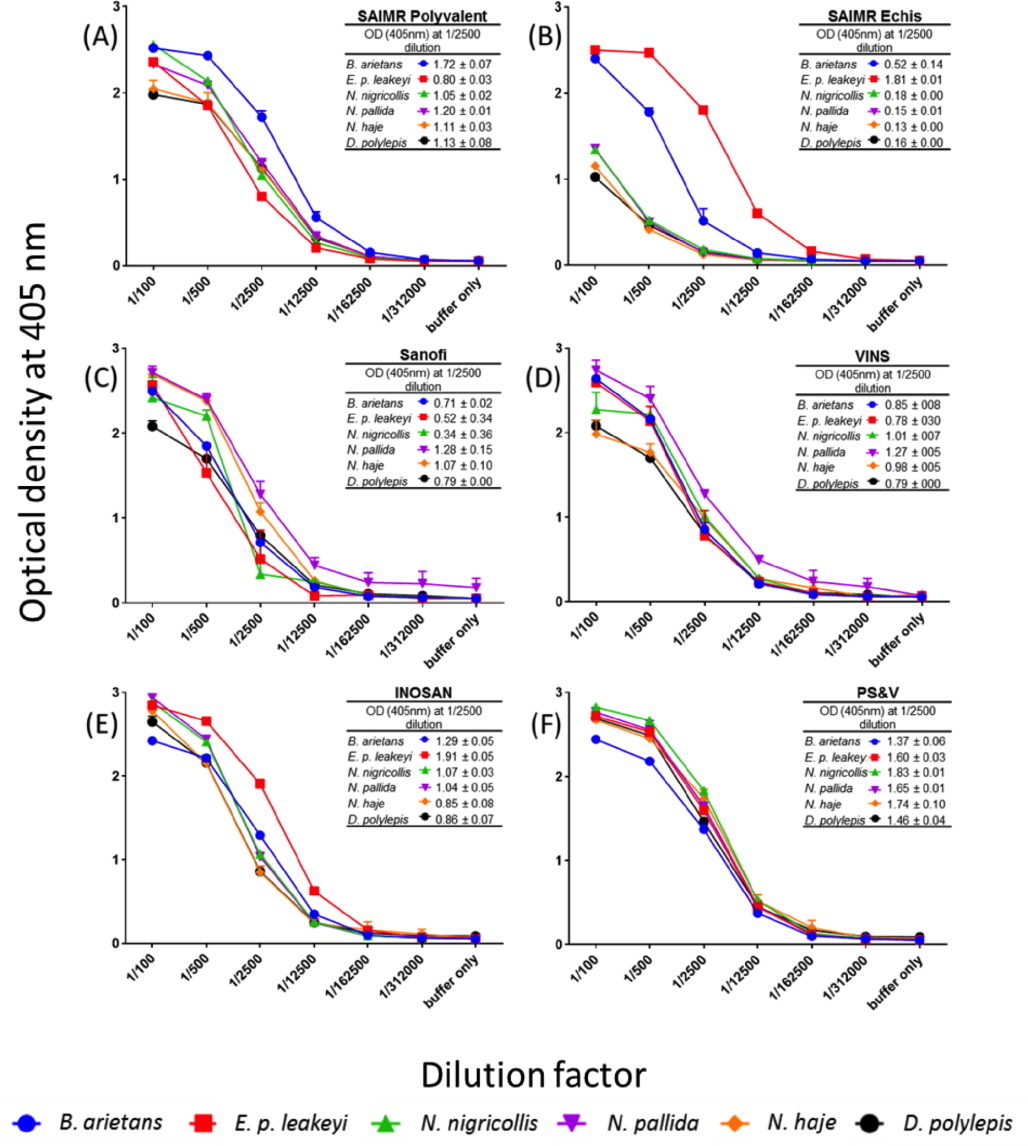
The IgG reactivity (titre) of six commercial antivenoms against six sub-Saharan Africa venoms determined by titration ELISA. All antivenoms were adjusted to 5 mg/ml in PBS prior to being diluted 1 in 100 and then serially diluted 1 in 5 and applied to venoms. Panel (A) = SAIMR polyvalent, (B) = SAIMR ECHIS CARINATUS, (C) = Sanofi Pasteur, (D) = VINS (E) = INOSAN, (F) = Premium Serums & Vaccines (PS&V) antivenoms. Venoms: *B*. *arietans* (blue), *E*. *p*. *leakeyi* (red), *N*. *nigricollis* (green), *N*. *pallida* (purple), *N*. *haje* (orange) and *D*. *polylepis* (black). Results are the mean of three replicates with error bars representing standard deviation (SD). Error bars are not shown where SD is smaller than data point. The reactivity of control, naïve horse IgG to each venom was consistently low at each dilution for each venom (mean OD 405 nm = 0.07 ± 0.005). For interested readers, the same data as in Fig 3 is presented in Supplementary S1 Fig to facilely compare binding to each of the six venoms.

To ensure valid cross-comparison of antivenom-venom reactivity, we first standardised the IgG concentration of each antivenom to 5 mg IgG/ml. We next incubated serial dilutions of each antivenom with the same concentration of each venom (Fig 3). For space reasons we have excluded the graph showing the baseline reactivity of the naive control horse IgG to all the venoms. The OD readings of the antivenoms at the 1:2,500 dilution, in the middle of the downward slope, provide the most immunologically meaningful comparison and, for clarity, are presented as tables in each panel of Fig 3. For example, at this dilution, the SAIMR ECHIS CARINATUS antivenom IgG shows (i) highest binding to the *E*. *p*. *leakeyi* venom, (ii) some cross-reactivity to other viper (puff adder) venom proteins, and (iii) near-zero binding to the four elapid venoms–results entirely consistent with an antivenom generated by immunisation with only *Echis* species venoms.

While detectable venom-binding differences between the antivenoms exist, none of the antivenoms exhibited sufficiently poor IgG binding to venoms that account for the very poor polyspecific ED_50_ results of, for example, the VINS and INOSAN antivenoms (Fig 2). Thus, at the same IgG dilution (1:2,500) the OD values (venom-binding) of VINS antivenom to all the venoms was greater or equivalent to that of the more preclinically effective Sanofi Pasteur antivenom. Furthermore, INOSAN’s inefficacy against *D*. *polylepis* venom contrasted with its higher OD to this venom than the considerably more efficacious Sanofi Pasteur antivenom. Finally, the OD values of the Premium Serums & Vaccines antivenom was consistently higher to all the venoms than the more efficacious SAIMR antivenoms.

The ELISA IgG titration assay demonstrated that all the ‘test’ antivenoms contained venom-binding IgG titres not dissimilar to the ‘gold standard’ SAIMR antivenoms. Thus, while we identified many discreet IgG-venom binding differences between the antivenoms, we were unable to confidently attribute any of these as being responsible for the very different venom-neutralisation efficacies of these antivenoms. We therefore next performed an assay to identify whether the antivenoms possessed IgGs of variable avidity (binding strength) to the six different venoms that matched their distinct venom-neutralising efficacies.

### Comparison of the avidity of antivenom F(ab')_2_ IgG binding to the East African snake venoms by a chaotropic ELISA assay

Ammonium thiocyanate (NH_4_SCN) is a potent disruptor of protein-protein binding (chaotrope) and, by measuring the ELISA OD readings of the same concentration of IgG and venom in the presence of increasing amounts of the chaotrope, is used to test antivenom IgG-venom protein binding strength.

**Fig 4:**
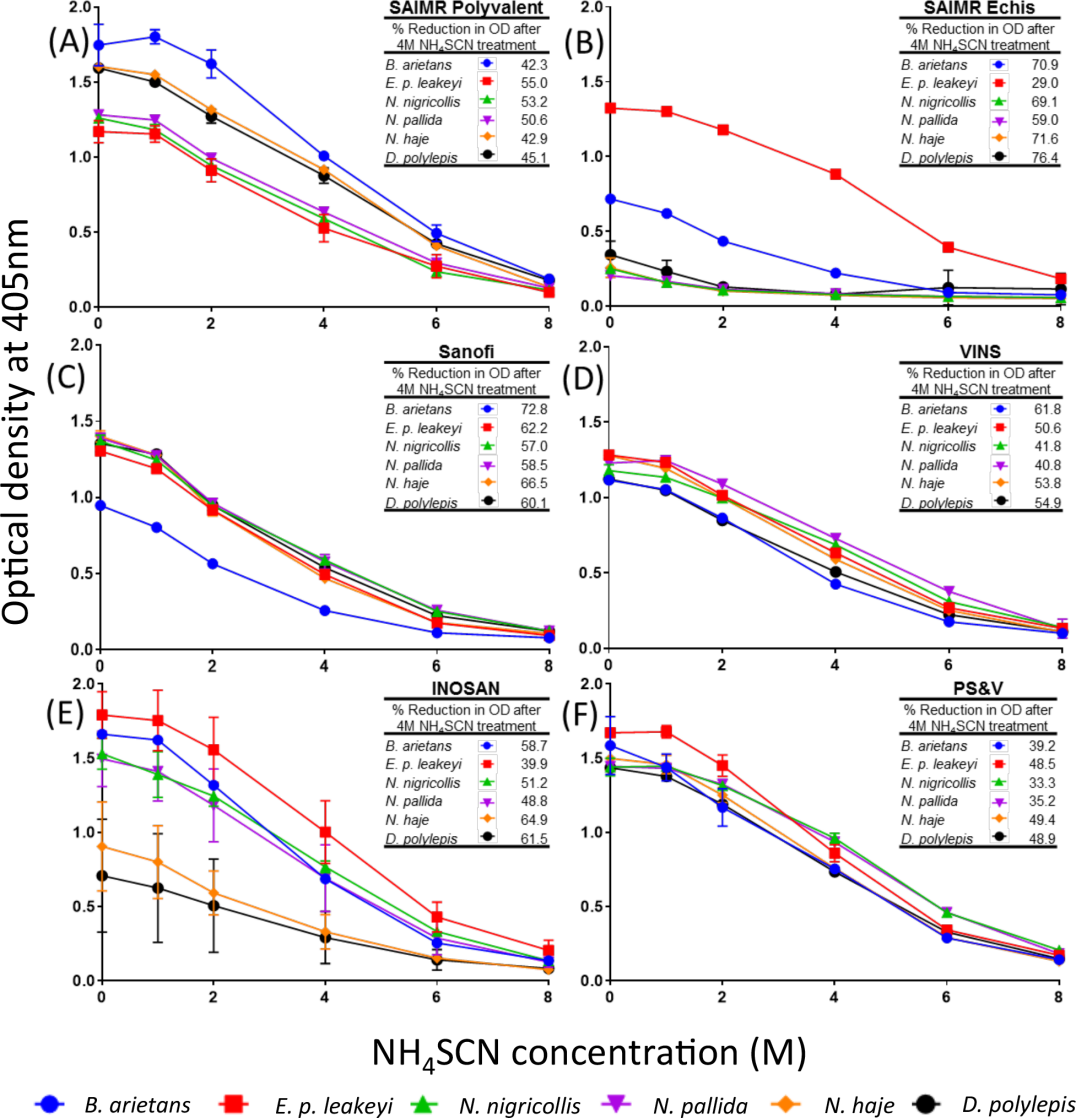
The relative IgG-venom binding avidity of six commercial antivenoms against six sub-Saharan Africa venoms determined by chaotropic ELISA. 1:1,000 dilutions of 5 mg/ml standardized antivenoms were allowed to bind to venom coated plates before being exposed to NH_4_SCN at increasing concentrations for 15 minutes. Panel (A) = SAIMR polyvalent, (B) = SAIMR ECHIS CARINATUS, (C) = Sanofi Pasteur, (D) = VINS (E) = INOSAN, (F) = Premium Serums & Vaccines (PS&V) antivenoms. Venoms: *B*. *arietans* (blue), *E*.*p*. *leakeyi* (red), *N*. *nigricollis* (green), *N*. *pallida* (purple), *N*. *haje* (orange) and *D*. *polylepis* (black). Results are the mean of three replicates with error bars representing standard deviation (SD). Error bars are not shown where SD is smaller than data point. The reactivity of control, naïve horse IgG to each venom was consistently low at each dilution for each venom (mean OD 405 nm = 0.05 ± 0.004). For interested readers, the same data as in Fig 4 is presented in Supplementary S2 Fig to facilely compare binding to each of the six venoms.

We incubated 1:1,000 dilutions of each of the 5 mg/ml standardised antivenom solutions with the venoms (in the same concentration as for the IgG titre ELISA) and determined the OD readings after addition of 0, 1, 2, 4, 6 and 8 moles of NH_4_SCN (Fig 4; we have excluded the graph showing the baseline reactivity of the naive control horse IgG to all the venoms). The most immunologically-informative results were gained by comparing the percentage reduction in OD values of the antivenoms without NH_4_SCN to that in the middle of the downward slope at 4 M NH_4_SCN—as illustrated by the table inserted into each panel of Fig 4.

This assay revealed that the Premium Serums & Vaccines (panel 4F) antivenom possesses the most consistent, and highest, cross-snake species IgG-venom binding avidity of all the antivenoms, including the ‘gold standard’ antivenoms. The INOSAN antivenom (panel 3E) exhibits the least consistent cross-species venom binding avidity.

The chaotropic ELISA assay revealed closer links between antivenom IgG-venom binding avidity and antivenom efficacy than the IgG titre results. Thus, the notably higher IgG-binding avidity of the SAIMR polyvalent gold standard (panel 4A) to the *B*. *arietans*, *N*. *haje* and *D*. *polylepis* venoms, and lower avidity to the spitting cobra (*N*. *nigricollis*, *N*. *pallida*) venoms accurately reflect the venom-neutralising dose-efficacy of this antivenom (Table 4). Also, the substantially higher IgG avidity of the INOSAN antivenom to *E*. *p*. *leakeyi* venom also matched its venom-neutralising dose-efficacy profile.

However, this assay did not provide data accounting for the snake-species distinct venom-neutralising efficacies of the Premium Serums & Vaccines, VINS and Sanofi Pasteur antivenoms, particularly in comparison with the superior venom-neutralising dose-efficacy of the SAIMR polyvalent antivenom against *N*. *haje*, *D*. *polylepis* and *B*. *arietans* venoms.

Venoms consist of multiple distinct protein groups and the protein composition of venom is markedly snake genus/species specific–with obvious implications on the venom protein-specificity of IgGs from venom-immunised horses. Neither the IgG titre nor the IgG avidity ELISA assays are designed to examine IgG binding to specific venom proteins. We therefore next used an immunoblot assay to investigate whether the six antivenoms express differences in IgG venom protein specificities that match their distinct venom-neutralising efficacies.

### Comparative analysis of antivenom IgG specificity to venom proteins of the East African snakes

**Fig 5:**
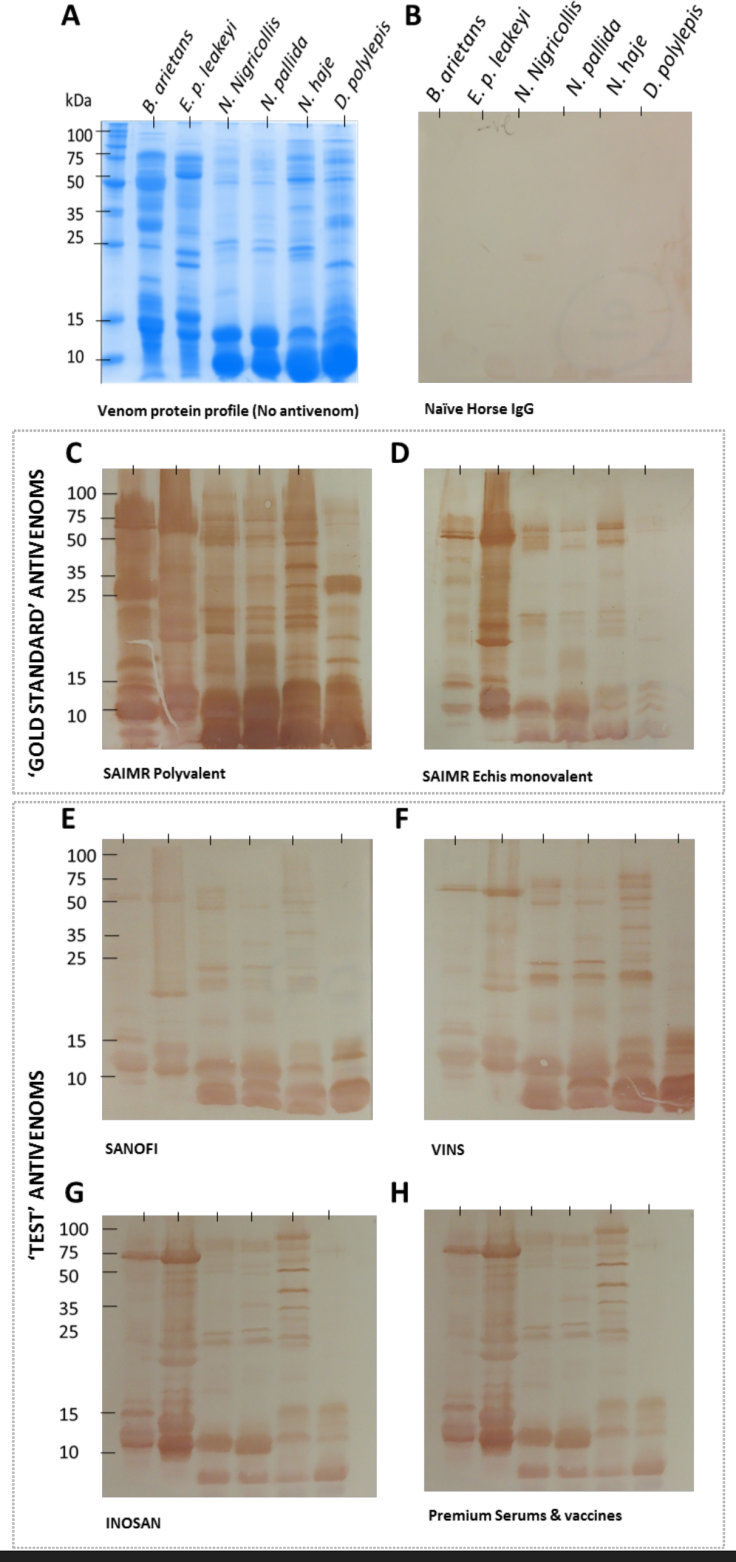
The IgG specificities of six commercial antivenoms to venom proteins of six sub-Saharan Africa venoms. Venoms (10 μg) of *B*. *arietans*, *E*. *p*. *leakeyi*, *N*. *nigricollis*, *N*. *pallida*, *N*. *haje* and *D*. *polylepis* were separated by reduced SDS-PAGE and visualised by coomassie blue staining (Panel A). Venom proteins in identical gels were transferred to nitrocellulose blots and incubated with 1:5,000 dilutions of naïve Horse IgG (B), the ‘gold standard’ SAIMR polyvalent (C) and SAIMR ECHIS CARINATUS (D) antivenoms, and the ‘test’ Sanofi Pasteur (E), VINS (F), INOSAN (G) and Premium Serums & Vaccines (H) antivenoms. The antivenoms were not standardised to 5 mg/ml as in the ELISA assays.

Fractionation of the six snake venoms by 15% SDS-PAGE revealed the numerical and molecular size diversity of the venom proteins (Fig 5A), with the cobra and mamba venoms (*N*. *nigricollis*, *N*. *pallida*, *N*. *haje* and *D*. *polylepis*) possessing a greater abundance of the low molecular mass neurotoxins/cytotoxins than the more evenly distributed molecular mass of the enzyme-rich, haemostasis-disruptive viper venoms (*E*. *p*. *leakeyi* and *B*. *arietans*). To determine the extent to which this wide spectrum of East African snake venom proteins are bound by IgG of the six antivenoms, we electrophoretically transferred these venom proteins to a membrane and incubated those (under identical conditions) with the antivenoms at 1:5,000 dilutions (Fig 5). For this test, we did not adjust the antivenoms to a standard 5 mg/ml concentration.

This analysis demonstrated that the intensity of the IgG-venom protein binding of the ‘gold standard’ SAIMR polyvalent and SAIMR ECHIS CARINATUS antivenoms was notably greater than all the ‘test’ antivenoms (Fig 5). It is important to note that, with minor brand-specific differences, that the difference between the ‘test’ and ‘gold standard’ antivenoms in this assay related to the intensity of venom protein binding and not the protein specificity. Thus, although the intensity of the IgG-venom protein binding was lower with the ‘test’ antivenoms, the immunoblots revealed that each antivenom possessed IgGs with similar venom protein specificities as the SAIMR ‘gold standard’ antivenoms. The lack of venom-reactivity of the control, naïve horse IgG (Fig 4B), evidences the venom-specificity of the antivenom IgGs.

The three immune assays above identified detectable differences in the IgG titre, avidity and venom-protein specificities of the six antivenoms, but these differences were relatively minor and could not be consistently applied to the interaction of each antivenom with each venom. We were therefore unable to identify an IgG-venom binding deficiency with any of the ‘test’ antivenoms, and by inference a deficiency in their venom-immunisation protocols, that could account for the ineffectiveness/weak efficacy of some of the ‘test’ antivenoms in our preclinical assays. The substantially higher IgG-venom protein binding intensity of the SAIMR polyvalent antivenom in the immunoblot assay suggested to us that this antivenom may be formulated with a higher amount of IgG/vial than the others.

Our final test was therefore to determine the protein (IgG) concentration (all antivenoms are formulated as F(ab’)_2_ fragments of IgG) of each antivenom because this has an obvious bearing on dose-efficacy, and because it was not stated by any manufacturer, despite it being the active component of these therapies.

### Comparative analysis of antivenom IgG content

We used a spectrophotometric instrument (NanoDrop) to measure protein content of each antivenom (in triplicate) and the results are presented in Table 5. We included control horse IgG of different known concentrations, to confirm the accuracy of the NanoDrop results. For the sake of completeness, we also used SDS-PAGE analysis of each antivenom to demonstrate their consistent IgG purity (Supplementary S3 Fig).

**Table 5:** The concentration of protein (F(ab’)_2_-fragment of IgG) in each vial of antivenom.

Antivenom Brand	mg/ml
PS&V—PAN AFRICA polyvalent	63.3 ± 6.2
VINS—Pan African	21.7 ± 2.4
INOSAN—Inoserp PANAFRICAIN	31.7 ± 6.2
Sanofi—FavAfrique	96.7 ± 12.5
SAVP—SAIMR Polyvalent	111.7 ± 27.2
SAVP—SAIMR Echis monovalent	71.7 ± 27.2

This analysis demonstrated the substantial inconsistency in the total IgG content of the antivenoms, and, importantly, that the VINS and INOSAN antivenoms respectively contained 19% and 28% of the IgG concentration of the SAIMR polyvalent antivenom. The IgG content of antivenom therefore exhibited the closest association to their comparative efficacy in neutralising the venoms of *N*. *nigricollis*, *N*. *pallida* and *E*. *p*. *leakeyi* (Fig 2). It is not however a universally-applicable explanation, because, for example, IgG content alone does not explain the superior anti-*B*. *arietans* and anti-*E*. *p*. *leakeyi* efficacy of Premium Serums & Vaccines (63 mg/ml) antivenom over that of the Sanofi Pasteur (96.7 mg/ml) antivenom. Assessing the antivenom efficacy data by mg antivenom did reveal that the anti-*E*. *p*. *leakeyi* venom efficacy of the INOSAN antivenom was equivalent to that of the SAIMR ECHIS CARINATUS ‘gold standard’ antivenom. Nevertheless, as stated above and depicted in Fig 2, it required 2.5 times more the volume of the SAIMR antivenom for the INOSAN product to achieve this efficacy parity, which in the human-treatment context has cost and adverse effect implications.

For completeness, we have presented the amount (mg) and volume (μl) of antivenom used for each of the ‘test’ antivenoms for each of the doses examined into Supplementary S1 Table. To facilitate comparison, we have also added the amount (mg) and volume (μl) of the calculated 2xED_50_ doses of the ‘gold standard’ antivenoms that provided 100% protection to the envenomed mice. This table therefore provides all the numerical data related to the preclinical assays, and interpretations of efficacy from this table is no different from that presented in Fig 2. No matter whether the data is examined by antivenom volume in μl or amount in mg, the least polyspecifically-effective antivenoms simply do not compare well to the ‘gold standard’ and to some of the other ‘test’ antivenoms. It was important that we tested the antivenoms by volume because that is the formulation in which the antivenom is provided by the manufacturer and used by the clinician. It is near impossible to envisage a clinician calculating the dose volume of antivenom he/she is going to administer based upon the mg/ml antibody content (and perhaps impossible because, as here, manufacturers rarely provide this information). Therefore, to ensure that our preclinical efficacy testing of the antivenoms is of value to clinicians and medicine-purchasing agencies in East Africa, we undertook this testing, and report the results by antivenom volume.

The reality is that the efficacy of a monospecific antivenom to its homologous venom is dictated by multiple factors, including IgG concentration, titre, avidity and protein specificity, which are themselves affected by the quality of the immunising venoms, the quantitative ratio of the venoms used for immunisation, the selected adjuvant and other aspects of the immunisation and antivenom-manufacturing protocols. All these interlocking factors are made more complex, as here, by increasing the number of venoms used to manufacture polyspecific antivenom, making it very difficult/impossible to confidently assign any one factor as being primarily responsible for lack of efficacy.

## Discussion

The plight of snakebite victims, particularly those in sub-Saharan Africa has been the subject of considerable recent attention [see 6, 8, 19, 20, 21] and the focus of recent Wellcome Trust and the Kofi Annan Foundation sponsored international meetings to identify remedial interventions. Reports from both meetings [4, 5] identify the urgent need for the provision of effective antivenom and preclinical efficacy testing of existing and new antivenoms to ensure this. This recommendation aligns fully with the new WHO initiative to establish a prequalification programme for African antivenom, which includes establishing venom standards for sub-Saharan Africa and using those for preclinical antivenom-efficacy testing [22]. Over the past five years, we have generated a comprehensive inventory of sub-Saharan African snake venoms of defined gene and protein composition and murine toxicity [23, 24, 25]. This unique resource has enabled the Kenya Snakebite Research & Intervention Centre to conduct this first preclinical assessment of the efficacy of antivenoms available for clinical use in Kenya.

This is important because the market failure of the Sanofi Pasteur antivenom and the very high costs of the SAIMR antivenoms leaves many East African countries with a choice of polyspecific antivenoms restricted to brands for which there is very little/no published data on their human effectiveness. Our preclinical results illustrate that the SAIMR polyvalent antivenom is considerably more effective in neutralising the murine lethality of the Egyptian cobra (*N*. *haje*), black mamba (*D*. *polylepis*) and puff adder (*B*. *arietans*) venoms than any of the ‘test’ antivenoms. The ‘test’ antivenoms exhibited a superior or equal dose-efficacy as the SAIMR polyvalent antivenom against the spitting cobra venoms (*N*. *nigricollis* and *N*. *pallida*). Preclinical neutralisation of the saw-scaled viper (*E*. *p*. *leakeyi*) venom was achieved by the Premium Serums & Vaccines and INOSAN antivenoms but required 2.5-fold greater volumes than the SAIMR ECHIS monovalent antivenom. Perhaps the most important result of our study was that no single antivenom, at the doses tested (see below for a detailed consideration of this assay), was effective in neutralising the murine lethal effects of venoms of all six medically important snakes of East Africa—despite the pan-African efficacy claims inherent in the names of many of these products. The snake species-specific dose efficacy of each of the ‘test’ and ‘gold standard’ SAIMR polyvalent antivenoms suggests that the clinical management of envenoming by these snakes with any one of these antivenoms may require distinct, snake species-specific dose regimens. In the absence of a rapid snake species diagnostic test, this is clinically problematic and will likely result in the administration of too little or too much antivenom–both highly undesirable scenarios resulting in either inefficacy or increased risk of antivenom-induced adverse effects, respectively.

On a more positive note, the preclinical efficacy of the Premium Serums & Vaccines product matched that of the highly regarded, but now unavailable Sanofi Pasteur product and approached that of the expensive SAIMR polyvalent antivenom. The Premium Serums & Vaccines product, at 26% of the cost of the SAIMR antivenoms (see Table 6), was the most affordable and effective antivenom of those tested here, although we note that preclinical efficacies against the two neurotoxic snake venoms (*N*. *haje* and *D*. *polylepis*) were weaker than against the other four species. Our results suggest that the preclinical efficacy of the VINS and INOSAN products could possibly be substantially improved by simply increasing the amount of IgG in each vial. The INOSAN product was the most expensive of the ‘test’ antivenoms, the most preclinically effective at neutralising the saw-scaled viper venom and one of the less effective antivenoms at neutralising the lethality of other snake venoms. It is notable that the IgG avidity of this antivenom to venoms of the six snakes varied more than the other antivenoms (Fig 4), and this avidity profile matched its snake-specific venom-neutralising efficacy. This may suggest that changes to the venom-immunisation regimen or analysis of the quality of the venoms could improve the venom-binding avidity and perhaps the efficacy of this antivenom. In consideration of the clinical efficacy of the SAIMR polyvalent and ECHIS monovalent antivenoms, it would be interesting to know whether SAVP has plans to combine the venom-immunising mixtures of these products to produce a truly pan-African polyspecific antivenom.

**Table 6:** Costs to Kenya hospitals of antivenoms (purchased through Mission for essential drugs & supplies (US$1 = Kshs 101.8; Feb 2016).

Antivenom Brand	US$/vial
PS&V—PAN AFRICA polyvalent	$83.5
VINS—Pan African	$47.9
INOSAN—Inoserp PANAFRICAIN	$105.1
Sanofi—FavAfrique	$99
SAVP—SAIMR Polyvalent	$315
SAVP—SAIMR Echis monovalent	$315

We have carefully qualified our interpretation/extrapolation of the results of our preclinical ‘gold standard’ comparison assays to the efficacy of antivenom treatment of human snakebite patients, and urge readers to be similarly cautious. Our preclinical protocol enables a rapid efficacy comparison of a matrix of six venoms and four antivenoms using the minimum number of mice, but it differs from the recommended WHO antivenom-efficacy testing protocols in that it does not provide an ED_50_ value for each ‘test’ antivenom against each venom. Thus for example, we are unable to state whether, or not, the polyspecifically effectiveness of the VINS and INOSAN products could attain 100% efficacy against more of the venoms by using substantially greater volumes–because of the volume constraints inherent to this murine assay. From a human-treatment perspective, the necessity to administer multiple vials of an antivenoms carries important treatment costs (the INOSAN product was the most expensive ‘test’ antivenom) and adverse effects issues that risk poor uptake in rural tropical regions.

On a more general note, these murine preclinical antivenom-efficacy testing assays are not infallible predictors of human efficacy. A recent study in Sri Lanka has questioned the value of predicting efficacy outcomes in human patients from ED_50_ results [26]. Conversely, the undoubted ability of the ED_50_ test to discriminate between effective and ineffective antivenoms in the murine model [13, 27], and the effective use of ED_50_ data [15] to help design the dose regimen of a human antivenom clinical trial [28] suggests that while the ED_50_ preclinical test has inadequacies many practitioners share, it should be retained until more accurate assays are tested, validated and become available. Thus, while the results of this study identify a potentially serious therapeutic concern, one of the priorities of the Kenya, Nigerian and Cameroon Snakebite Research & Intervention Centres will be to undertake conventional ED_50_ tests so that the Ministries of Health can be provided with pharmacopoeia-compliant data that they can act upon to restrict human use of preclinically-ineffective antivenom.

The dialogue above underscores the urgent need for more published data on the efficacy of the different antivenom brands in use in sub-Saharan Africa to treat human patients. The paucity of this information reflects the prohibitive expense, problems of recruiting sufficient patients envenomed by the myriad of venomous snake species, the lack of accurate diagnostic tools to distinguish such species-distinct envenoming, and time required to conduct full clinical trials. Until the funding and required clinical/diagnostic tools become available, we remain reliant upon clinical observation studies that have importantly identified the wide-spread use of some dangerously ineffective antivenoms [9–11]. However, only the first of these reports provided the clinically-vital information on the antivenom brand. Another objective of the recently-established African Snakebite Research Group will be to conduct surveys in many rural hospitals in Nigeria, Kenya and Cameroon experiencing high snakebite admissions. The outcomes of these surveys will include reporting antivenom availability, and assessments of the clinical outcomes of treating patients with the various brands of locally-available antivenom.

In conclusion, this first report of the African Snakebite Research Group identifies a worrying differential in the preclinical efficacy of available antivenoms in Kenya and underscores the need for independent preclinical testing of antivenoms throughout sub-Saharan Africa, and the need for venom standards for all the most medically important snakes to resource this testing. There is also an urgent need for practitioners to publish data on the clinical outcomes of treating patients with brand-named antivenoms (despite the inherent problem of such transparency). The widespread availability of pan-African preclinical testing and clinical observation information will substantially help to improve the effectiveness of snakebite management and thereby reduce the high case fatality currently suffered by sub-Saharan African snakebite victims.

## Supporting information

S1 Fig The same results as manuscript Fig 3 displayed by venom. Titration ELISA of six commercial antivenoms, standardised to 5mg/ml, against six East African venoms. All antivenoms were adjusted to 5 mg/ml in PBS prior to being diluted 1:100 and then serially diluted 1:5 and applied to venoms. Panel (A) = *B*. *arietans*, (B) = *E*.*p*. *leakeyi*, (C) = *N*. *nigricollis*, (D) = *N*. *pallida*, (E) = *N*. *haje*, (F) = *D*. *polylepis*. Antivenoms: SAIMR polyvalent (blue), SAIMR ECHIS (red), Sanofi Pasteur (green), VINS (purple), INOSAN (orange), Premium Serums & Vaccines (PS&V, black) and naïve equine IgG (brown). Results are the mean of three replicates with error bars representing standard deviation (SD). Error bars are not shown where SD is smaller than the data point.
(DOCX)

S2 Fig The same results as manuscript Fig 4 displayed by venom. The relative IgG-venom binding avidity of six commercial antivenoms against six East African venoms determined by chaotropic ELISA. 1:1000 dilutions of standardized (5 mg/ml) antivenoms were allowed to bind to venom coated plates before being exposed to NH_4_SCN at increasing concentrations for 15 minutes. Panel (A) = *B*. *arietans*, (B) = *E*. *p*. *leakeyi*, (C) = *N*. *nigricollis*, (D) = *N*. *pallida* (E) = *N*. *haje*, (F) = *D*. *polylepis*. Venoms: SAIMR polyvalent (blue), SAIMR Echis (red), Sanofi (green), INOSAN (purple), VINS (orange), PS&V (black) and naïve equine IgG (brown). Results are the mean of three replicates with error bars representing standard deviation (SD). Where SD is smaller than data point error bars are not shown.
(DOCX)

S3 Fig 15% SDS-PAGE gels visualising antivenom protein profiles of the 6 antivenoms used in this study. Each well contains 10 μl of a 1 in 200 dilution of each antivenom. Panel A = non-reduced protein profile, panel B = reduced protein profile.
(DOCX)

S1 Table The comparative efficacy of the ‘test’ antivenoms against the East African snake venoms, described by the amount (mg) and volume (μl) of antivenom administered at volumes equivalent half (0.5 x), equal (1 x) or two and half times (2.5 x) of the dose of the comparable SAIMR ‘gold standard’ that protected 100% (the calculated ED_100_) of the mice from the lethal toxicity of the East African venoms. * Mice died from the high density of antivenom/venom complexes, not from venom-induced effects. This occurs occasionally in murine preclinical testing as a consequence of the 30 minute, 37°C incubation of the venom/antivenom mixture prior to injection. It likely has no clinical relevance, but can obfuscate preclinical results. ** This 2xED_50_ figure was calculated (double that) from the ED_50_ figure provided in Table 4. ND–not done. Blue boxes identify ‘test’ antivenoms & doses providing 100% protection against envenoming with lower amounts, mg, of antivenom (more dose-effective) than the 2xED_50_ ‘gold standard’ antivenom dose. Green boxes identify ‘test’ antivenoms & doses providing 100% protection against envenoming with higher amounts, mg, of antivenom (less dose-effective) than the 2xED_50_ ‘gold standard’ antivenom dose. Unshaded boxes identify antivenom & doses that failed to impart 100% protection to envenoming.
(DOCX)
