# Correction: Preclinical antivenom-efficacy testing reveals potentially disturbing deficiencies of snakebite treatment capability in East Africa

**DOI:** 10.1371/journal.pntd.0008698

**Published:** 2020-08-31

**Authors:** Robert A. Harrison, George O. Oluoch, Stuart Ainsworth, Jaffer Alsolaiss, Fiona Bolton, Ana-Silvia Arias, José-María Gutiérrez, Paul Rowley, Stephen Kalya, Hastings Ozwara, Nicholas R. Casewell

Fig 5 in this article [1] includes an error, in that the INOSAN data (panel G) were duplicated in panel H. A corrected version of Fig 5 is provided here.

**Fig 5:**
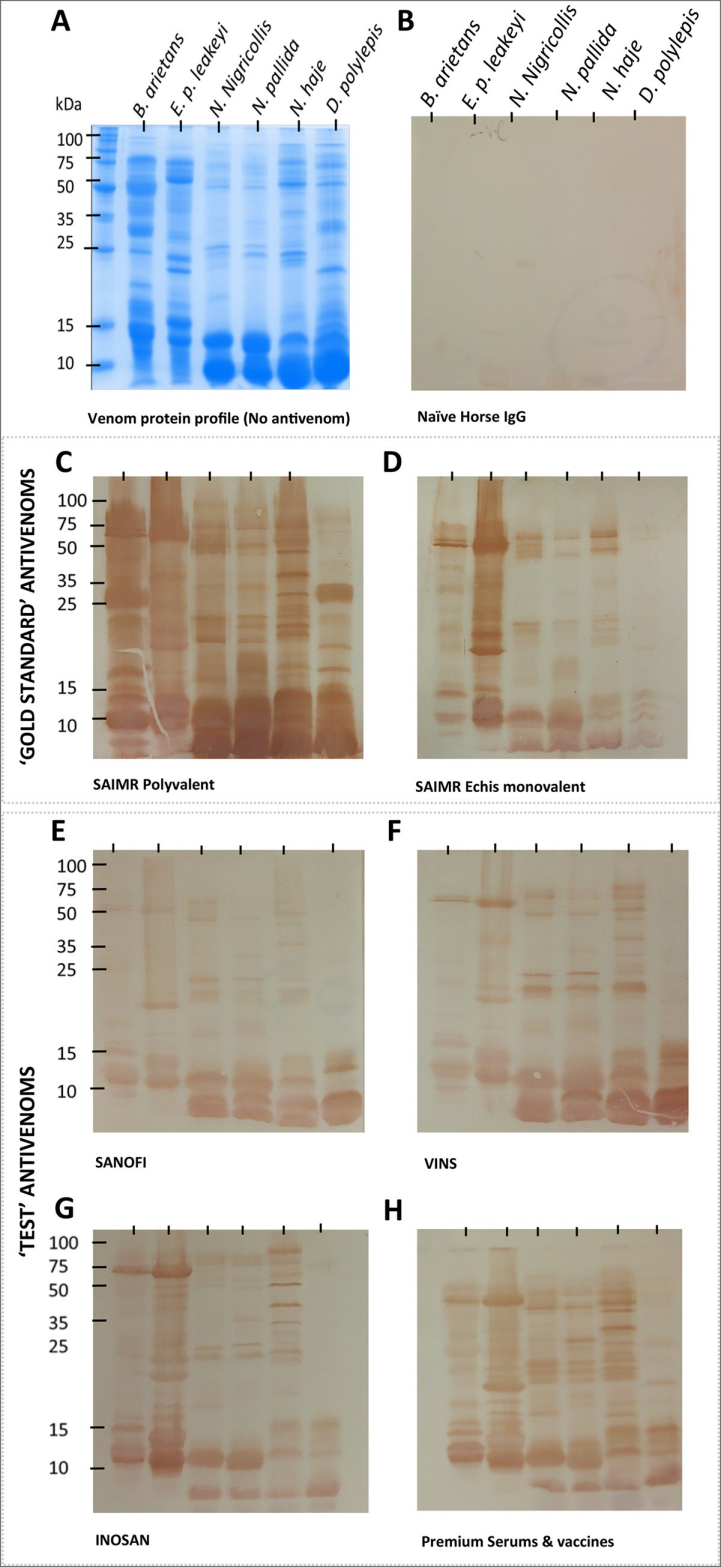
The IgG specificities of six commercial antivenoms to venom proteins of six sub-Saharan Africa venoms. Venoms (10 μg) of *B*. *arietans*, *E*. *p*. *leakeyi*, *N*. *nigricollis*, *N*. *pallida*, *N*. *haje* and *D*. *polylepis* were separated by reduced SDS-PAGE and visualised by coomassie blue staining (Panel A). Venom proteins in identical gels were transferred to nitrocellulose blots and incubated with 1:5,000 dilutions of naïve Horse IgG (B), the ‘gold standard’ SAIMR polyvalent (C) and SAIMR ECHIS CARINATUS (D) antivenoms, and the ‘test’ Sanofi Pasteur (E), VINS (F), INOSAN (G) and Premium Serums & Vaccines (H) antivenoms. The antivenoms were not standardised to 5 mg/ml as in the ELISA assays.

In addition, the underlying data were not included with the original published article, and are provided here in S1 Table.

The authors apologize for the error in the published article.

## Supporting information

S1 Table Raw ELISA data supporting results reported in Figs 3 and 4 of [1]. (XLSX)

## References

[1] Harrison RA, Oluoch GO, Ainsworth S, Alsolaiss J, Bolton F, Arias A-S, et al (2017) Preclinical antivenom-efficacy testing reveals potentially disturbing deficiencies of snakebite treatment capability in East Africa. PLoS Negl Trop Dis 11(10): e0005969 10.1371/journal.pntd.0005969 29045429 PMC5646754

