# Supplementary material for: Preclinical antivenom-efficacy testing reveals potentially disturbing deficiencies of snakebite treatment capability in East Africa
Source: PLoS Negl Trop Dis. 2017 Oct 18;11(10):e0005969. doi: 10.1371/journal.pntd.0005969 (PMC5646754; doi:10.1371/journal.pntd.0005969)

# Supplementary Figure 1

**Supplementary Figure 1**. **Same results as manuscript Figure 3 displayed by venom**. Titration ELISA of six commercial antivenoms, standardised to 5mg/ml, against 6 sub-Saharan Africa venoms. All antivenoms were adjusted to 5 mg/ml in PBS prior to being diluted 1:100 and then serially diluted 1:5 and applied to venoms. Panel (A) = *B. arietans,* (B) = *E.p. leakeyi*, (C) = *N. nigricollis*, (D) = *N. pallida,* (E) = *N. haje*, (F) = *D. polylepis.* Antivenoms: SAIMR polyvalent (blue), SAIMR ECHIS (red), Sanofi Pasteur (green), VINS (purple), INOSAN (orange), Premium Serums & Vaccines (PS&V, black) and naïve equine IgG (brown). Results are the mean of three replicates with error bars representing standard deviation (SD). Error bars are not shown where SD is smaller than the data point.

Optical density at 405 nm

Dilution factor


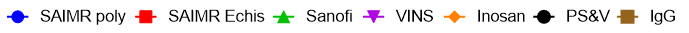

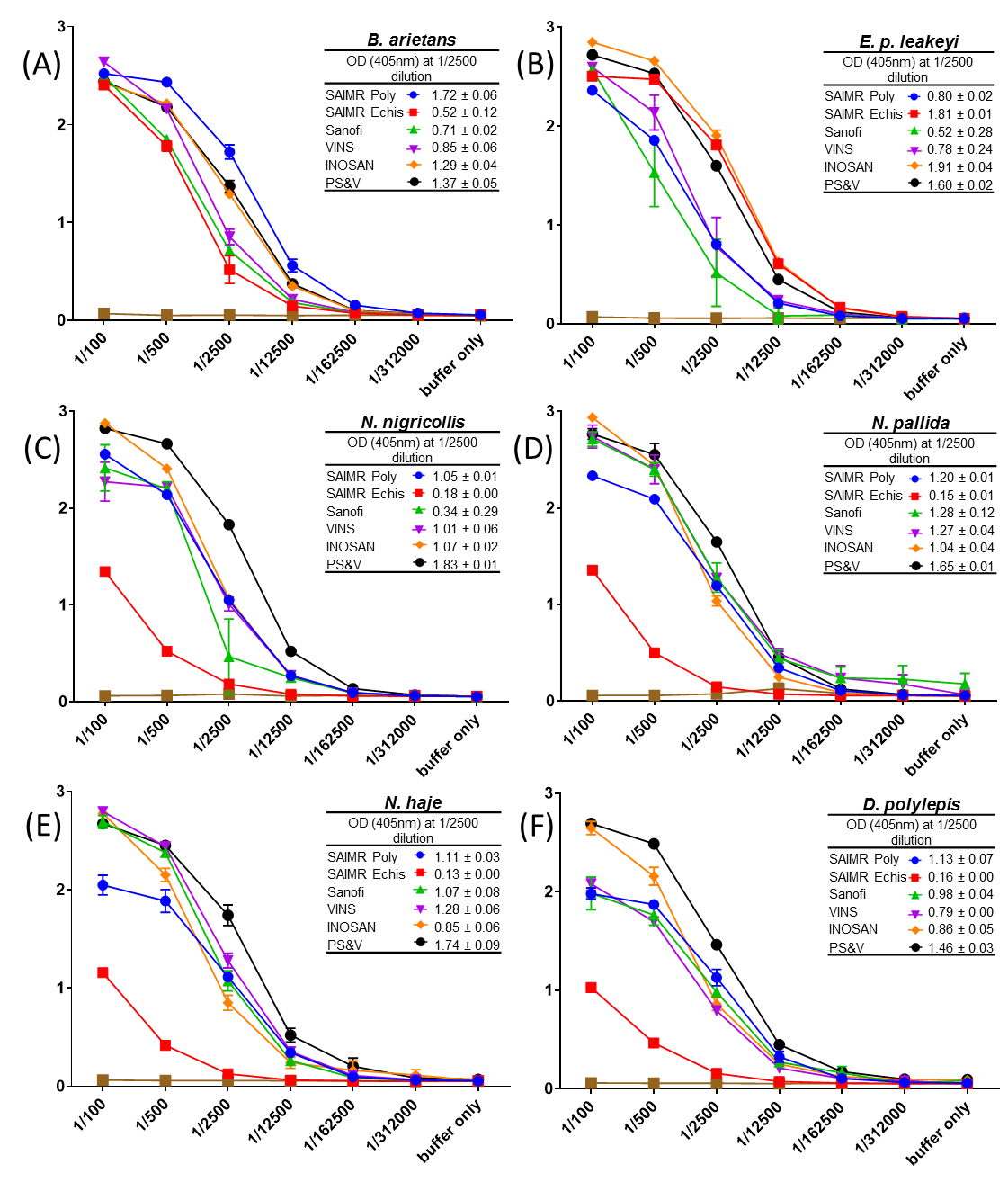

Supplement: S1 Fig — Titration ELISA of six commercial antivenoms, standardised to 5mg/ml, against six East African venoms. All antivenoms were adjusted to 5 mg/ml in PBS prior to being diluted 1:100 and then serially diluted 1:5 and applied to venoms. Panel (A) = B. arietans, (B) = E.p. leakeyi, (C) = N. nigricollis, (D) = N. pallida, (E) = N. haje, (F) = D. polylepis. Antivenoms: SAIMR polyvalent (blue), SAIMR ECHIS (red), Sanofi Pasteur (green), VINS (purple), INOSAN (orange), Premium Serums & Vaccines (PS&V, black) and naïve equine IgG (brown). Results are the mean of three replicates with error bars representing standard deviation (SD). Error bars are not shown where SD is smaller than the data point. (DOCX) [file pntd.0005969.s001.docx]
