# Supplementary figures and images for: Preclinical antivenom-efficacy testing reveals potentially disturbing deficiencies of snakebite treatment capability in East Africa
Source: PLoS Negl Trop Dis. 2017 Oct 18;11(10):e0005969. doi: 10.1371/journal.pntd.0005969 (PMC5646754; doi:10.1371/journal.pntd.0005969)

# Supplementary Figure 2


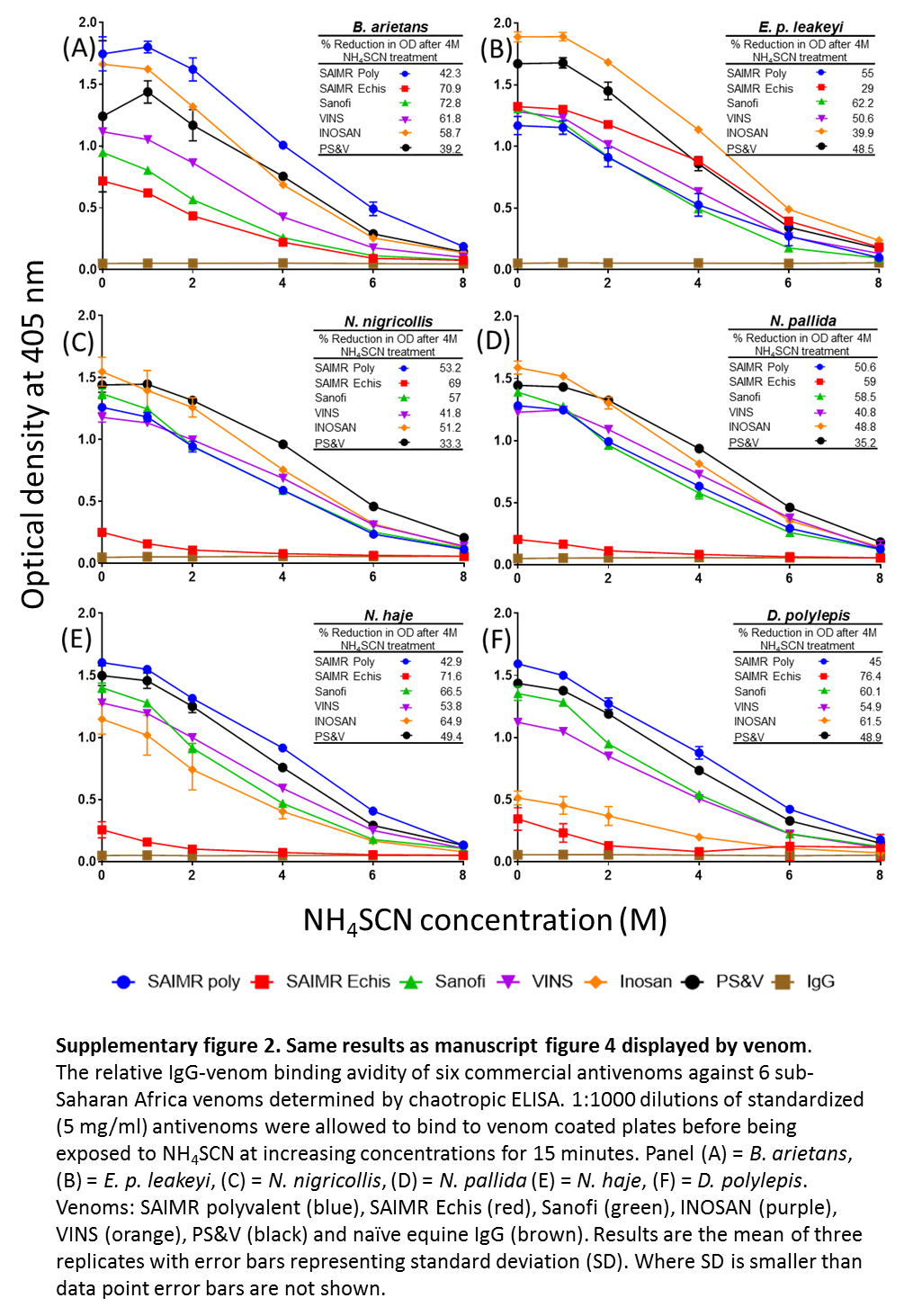

Supplement: S2 Fig — The relative IgG-venom binding avidity of six commercial antivenoms against six East African venoms determined by chaotropic ELISA. 1:1000 dilutions of standardized (5 mg/ml) antivenoms were allowed to bind to venom coated plates before being exposed to NH4SCN at increasing concentrations for 15 minutes. Panel (A) = B. arietans, (B) = E. p. leakeyi, (C) = N. nigricollis, (D) = N. pallida (E) = N. haje, (F) = D. polylepis. Venoms: SAIMR polyvalent (blue), SAIMR Echis (red), Sanofi (green), INOSAN (purple), VINS (orange), PS&V (black) and naïve equine IgG (brown). Results are the mean of three replicates with error bars representing standard deviation (SD). Where SD is smaller than data point error bars are not shown. (DOCX) [file pntd.0005969.s002.docx]

# Supplementary Figure 3

#
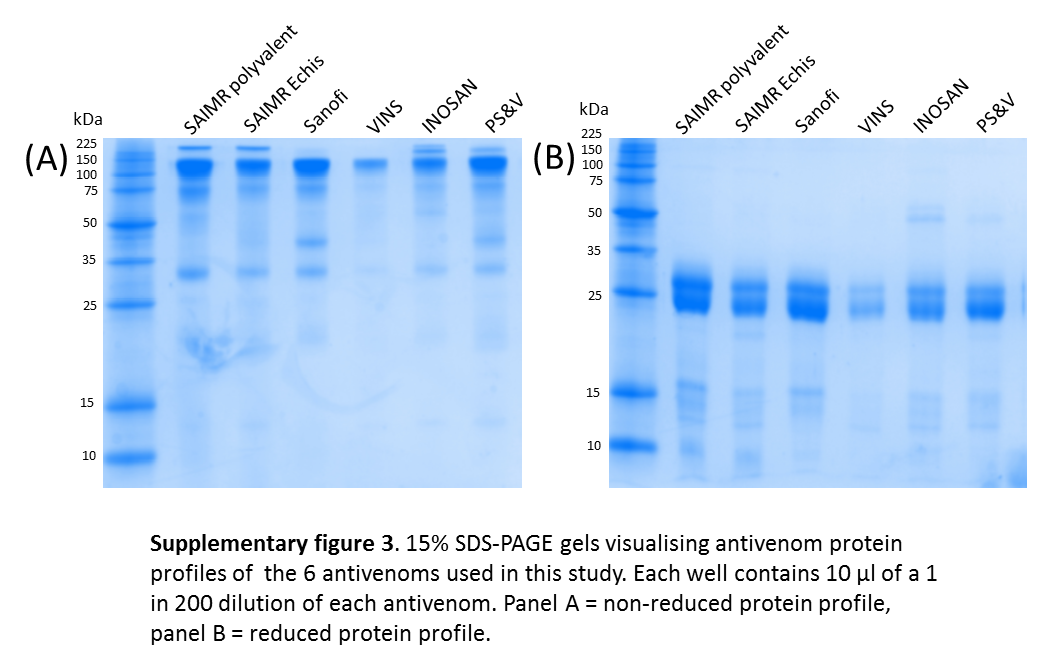

Supplement: S3 Fig — Each well contains 10 μl of a 1 in 200 dilution of each antivenom. Panel A = non-reduced protein profile, panel B = reduced protein profile. (DOCX) [file pntd.0005969.s003.docx]
