# Supplementary material for: Preclinical antivenom-efficacy testing reveals potentially disturbing deficiencies of snakebite treatment capability in East Africa
Source: PLoS Negl Trop Dis. 2017 Oct 18;11(10):e0005969. doi: 10.1371/journal.pntd.0005969 (PMC5646754; doi:10.1371/journal.pntd.0005969)
Supplement: S1 Table — * Mice died from the high density of antivenom/venom complexes, not from venom-induced effects. This occurs occasionally in murine preclinical testing as a consequence of the 30 minute, 37°C incubation of the venom/antivenom mixture prior to injection. It likely has no clinical relevance, but can obfuscate preclinical results. ** This 2xED50 figure was calculated (double that) from the ED50 figure provided in Table 4. ND–not done. Blue boxes identify ‘test’ antivenoms & doses providing 100% protection against envenoming with lower amounts, mg, of antivenom (more dose-effective) than the 2xED50 ‘gold standard’ antivenom dose. Green boxes identify ‘test’ antivenoms & doses providing 100% protection against envenoming with higher amounts, mg, of antivenom (less dose-effective) than the 2xED50 ‘gold standard’ antivenom dose. Unshaded boxes identify antivenom & doses that failed to impart 100% protection to envenoming. (DOCX) [file pntd.0005969.s004.docx]

**Supplementary Table 1.** The efficacy of the ‘test’ antivenoms (described by the amount (mg) and volume (μl) of antivenom administered at volumes equivalent half (0.5 x), equal (1 x) or two and half times (2.5 x) of dose of the SAIMR ‘gold standard’ antivenoms that protected 100% (the calculated 2xED_50_) of the mice from the lethal toxicity of the East African snake venoms.

|  | **The % survival of mice and the amount (mg) and volume (μl) of ‘test’ antivenoms examined at 0.5, 1 and 2.5 fold volumes of the SAIMR antivenoms that imparted 100% protection against envenoming** | | | | | | | | | | | | **The amount (mg) and volume (μl) of ‘gold standard’ SAIMR antivenoms imparting 100% protection (2xED_50_ dose) to mice **** | |
| --- | --- | --- | --- | --- | --- | --- | --- | --- | --- | --- | --- | --- | --- | --- |
|  | **Premium Serums & Vaccines PAN AFRICA** | | | **VINS**  **African** | | | **INOSAN**  **Inoserp PANAFRICAIN** | | | **Sanofi Pasteur**  **FavAfrique** | | |  |  |
| **Venom dose, μg (# LD_50_)** | **0.5 x** | **1x** | **2.5x** | **0.5 x** | **1x** | **2.5x** | **0.5 x** | **1x** | **2.5x** | **0.5 x** | **1x** | **2.5x** | **SAIMR polyvalent** | **SAIMR ECHIS** |
| *B. arietans* 97.8 (5 LD_50_) | 0 %  1.32 mg  21 μl | 40 %  2.65 mg  42 μl | 40 % *  6.64 mg  105 μl | 0 %  0.45 mg  21 μl | 0 %  0.91 mg  42 μl | 0 %  2.27 mg  105 μl | 0 %  0.66 mg  21 μl | 0 %  1.33 mg  42 μl | 40 %  3.32 mg  105 μl | 0 %  2.03 mg  21 μl | 0 %  4.06 mg  42 μl | 80 %  10.15 mg  105 μl | 100%  4.71 mg  42.14 μl |  |
| *E. p. leakeyi* 80.0 (5 LD_50_) | ND | 80 %  2.21 mg  35 μl | 60 % *  5.57 mg  88 μl | ND | 0 %  0.75 mg  35 μl | 20 %  1.90 mg  88 μl | ND | 60 %  1.11 mg  35 μl | 100 %  2.79 mg  88 μl | ND | 0 %  3.38 mg  35 μl | 80 %  8.51 mg  88 μl |  | 100%  2.52 mg  35.12 μl |
| *N. nigricollis* 61.0 (2.5 LD_50_) | 100 %  4.36 mg  69 μl | 100 %  8.73 mg  138 μl | ND | 0 %  1.49 mg  69 μl | 100 %  2.99 mg  138 μl | ND | 0 %  2.19 mg  69 μl | 100 %  4.37 mg  138 μl | ND | 100 %  6.67 mg  69 μl | 100 %  13.34 mg  138 μl | ND | 100%  15.45 mg  138.34 μl |  |
| *N. pallida*  46.5 (5 LD_50_) | 100 %  4.68 mg  74 μl | 100 %  9.37 mg  148 μl | ND | 0 %  1.61 mg  74 μl | 0 %  3.21 mg  148 μl | ND | 0 %  2.35 mg  74 μl | 100 %  4.69 mg  148 μl | ND | 100 %  7.16 mg  74 μl | 100 %  14.31 mg  148 μl | ND | 100%  16.42 mg  147.04 μl |  |
| *N. haje*  40.8 (5 LD_50_) | 0 %  4.49 mg  71 μl | 0 %  8.98 mg  142 μl | ND | 0 %  1.54 mg  71 μl | 0 %  3.08 mg  142 μl | ND | 0 %  2.25 mg  71 μl | 0 %  4.50 mg  142 μl | ND | 0 %  6.86 mg  71 μl | 20 %  13.73 mg  142 μl | ND | 100%  15.86 mg  142.0 μl |  |
| *D. polylepis* 30.8 (5 LD_50_) | 0 %  0.89 mg  14 μl | 0 %  1.77 mg  28 μl | 100 %  4.43 mg  70 μl | 0 %  0.30 mg  14 μl | 0 %  0.61 mg  28 μl | 0 %  1.52 mg  70 μl | 0 %  0.44 mg  14 μl | 0 %  0.89 mg  28 μl | 0 %  2.22 mg  70 μl | 0 %  1.35 mg  14 μl | 60 %  2.71 mg  28 μl | 100 %  6.77 mg  70 μl | 100%  3.08 mg  27.64 μl |  |
| * Mice died from the high density of antivenom/venom complexes, not from venom-induced effects. This occurs occasionally in murine preclinical testing as a consequence of the 30 minute, 37^o^C incubation of the venom/antivenom mixture prior to injection. It likely has no clinical relevance, but can obfuscate preclinical results. ** This 2xED_50_ figure was calculated (double that) from the ED_50_ figure provided in Table 4. ND – not done. Blue boxes identify ‘test’ antivenoms & doses providing 100% protection against envenoming with lower amounts, mg, of antivenom (more dose-effective) than the 2xED_50_ ‘gold standard’ antivenom dose. Green boxes identify ‘test’ antivenoms & doses providing 100% protection against envenoming with higher amounts, mg, of antivenom (less dose-effective) than the 2xED_50_ ‘gold standard’ antivenom dose. Unshaded boxes identify antivenom & doses that failed to impart 100% protection to envenoming. | | | | | | | | | | | | | | |
